# Phosphorylation-driven conformational switching of the ArnA–ArnB complex involved in archaeal motility regulation

**DOI:** 10.3389/fmicb.2025.1717585

**Published:** 2026-01-15

**Authors:** Mohamed Watad, Lukas Korf, Wieland Steinchen, Filipp Bezold, Marian S. Vogt, Po Hsun Wang, Leon Selbach, Sebastian Hepp, Luis Gayermann, Marleen van Wolferen, Xing Ye, Sonja-Verena Albers, Lars-Oliver Essen

**Affiliations:** 1 Department of Chemistry, Philipps University of Marburg, Marburg, Germany; 2 Center for Synthetic Microbiology, Philipps University of Marburg, Marburg, Germany; 3 Institute for Biology, Molecular Biology of Archaea, University of Freiburg, Freiburg, Germany; 4 University of Freiburg, Signalling Research Centres BIOSS and CIBSS, Freiburg, Germany

**Keywords:** archaea, ArnB, motility, order to disorder, regulation, sequential phosphorylation, starvation

## Abstract

ArnA and ArnB serve as regulators within the Sulfolobus archaellum regulatory network by modulating the archaellum components ArlB and ArlX, which are essential for swimming motility. Together, they form a dynamic complex that, depending on nutrient availability, exists in either a loose, unphosphorylated or a tight, phosphorylated state. This transition is directed by phosphorylation via the kinase ArnC. To investigate this transition, we determined the cocrystal structure of the ArnA/ArnB complex, revealing that the zinc finger domain of ArnA interacts with both the β-sandwich and the C-terminal domains of ArnB. HDX data support the phosphorylation-dependent transition from a loose to a tight ArnAB complex driven by sequential phosphorylation of ArnB. This modification exposes the interaction surface of the C-terminal domain of ArnB, which then binds to the forkhead-associated domain of ArnA. Upon starvation of deletion strains of *arnA* and *arnB*, a reduction of ArnA was observed in the Δ*arnB* strain, and a reduction in ArnB levels was seen in the Δ*arnA* strain. Additionally, several putative transcriptional regulators were affected, suggesting downstream regulatory effects. These results highlight the critical role of the ArnAB complex in regulating the archaellum response to nutrient cues and provide new insights into the complex regulatory network governing archaeal swimming motility.

## 1 Introduction

Reversible protein phosphorylation is a central regulatory principle in Archaea, where Ser/Thr/Tyr phosphorylation modulates metabolic adaptation, stress responses, and differentiation via Hanks-type kinases and PP2A-like phosphatases (Kennelly, 2014; Esser et al., 2016; Stancik et al., 2018). In *Sulfolobus acidocaldarius*, motility is governed by the archaellum regulatory network (arn), which integrates environmental signals, particularly nutrient availability, into transcriptional and post-transcriptional control of archaellum genes (Hoffmann et al., 2016, 2019). This complex regulatory network includes several transcriptional and post-translational regulators including ArnR, ArnR1, AbfR1 and ArnS, respectively (Haurat et al., 2017; Li et al., 2017; Bischof et al., 2019). Phosphorylation plays a central role in modulating the activity of many of these factors, the network therefore also depends on Ser/Thr phosphatase PP2A and kinases ArnC and ArnD (Reimann et al., 2013; Hoffmann et al., 2016). Central to this network are the FHA-domain protein ArnA and its partner ArnB, whose phosphorylation-dependent interaction resembles bacterial and eukaryotic FHA–pThr signaling systems that control cell like signal transduction and DNA-damage responses (Mahajan et al., 2008; Reimann et al., 2012; Christian Reinhardt and Yaffe, 2013).

The archaellum is the primary motility structure in Archaea, facilitating self-locomotion and enabling adaptive habitat transitions (Jarrell and Albers, 2012). In *S. acidocaldarius*, ArnA and ArnB act as negative regulators of arlB (archaellin) expression in response to environmental stimuli. Their tight complex formation during nutrient-rich conditions represses archaellum assembly, whereas nutrient limitation triggers complex dissociation, arlB upregulation, and hypermotility (Lassak et al., 2012, 2013; Reimann et al., 2012; Hoffmann et al., 2016, 2019). ArnA recognizes phosphorylated threonine residues, primarily T343 and T353, in the C-terminal four-helix bundle of ArnB. Besides this regulatory helical bundle (Hoffmann et al., 2019) ArnB consists of an N-terminal vWA domain and a topologically inserted β-sandwich domain. Phosphorylation and dephosphorylation of ArnB have been reported to proceed by the kinase ArnC and phosphatase PP2A, respectively (Ye et al., 2020). The down-stream mechanism of ArnAB-mediated transcription control has not yet been elucidated, although prior work in *S. islandicus* suggested a role as transcription factor for the ArnAB complex via the phosphorylation state of ArnB (Jiang et al., 2023).

Our previous structural studies resolved the FHA domain of ArnA (PDB: 5A8I) and ArnE (PDB: 5A8J), which is a paralog of ArnB, previously named as vWA2, but shows no phenotype upon deletion on motility like ArnB (Reimann et al., 2012; Hoffmann et al., 2019). However, the ArnA–ArnB interface and the precise role of ArnB phosphorylation as well as how it may affect downstream signaling remained unclear (Hoffmann et al., 2016, 2019). Mutation of the pThr sites T343 and T353 neither showed a hypermotile phenotype nor impaired complex formation, indicating that these potential phosphorylation sites may be not sufficient to disrupt ArnA–ArnB interaction. In contrast, deletion of the entire regulatory helical bundle (RHB) disrupted ArnAB complex formation and derepressed archaellum expression (Reimann et al., 2013; Hoffmann et al., 2019). Despite these insights, the molecular mechanism by which the ArnAB complex regulates *arlB* levels remains enigmatic.

In this study, we determined the structure of ArnB and characterized its phosphorylation-dependent interaction with ArnA. Our results reveal a sequential, phosphorylation-driven conformational switch in ArnB that modulates ArnA binding and functions as a nutrient-sensitive regulatory switch for archaellum expression. In the unphosphorylated state, we found loose ArnAB complex formation via the N-terminal zinc-finger domain of ArnA by co-crystallization and small-angle X-ray scattering (SAXS). Proteomic analysis of ∆*arnA* and ∆*arnB* strains further identified downstream targets, highlighting the broader regulatory scope of the ArnAB module in archaeal motility and adaptation.

## 2 Results

### 2.1 Overall structure of the unphosphorylated ArnAB complex

**Figure 1:**
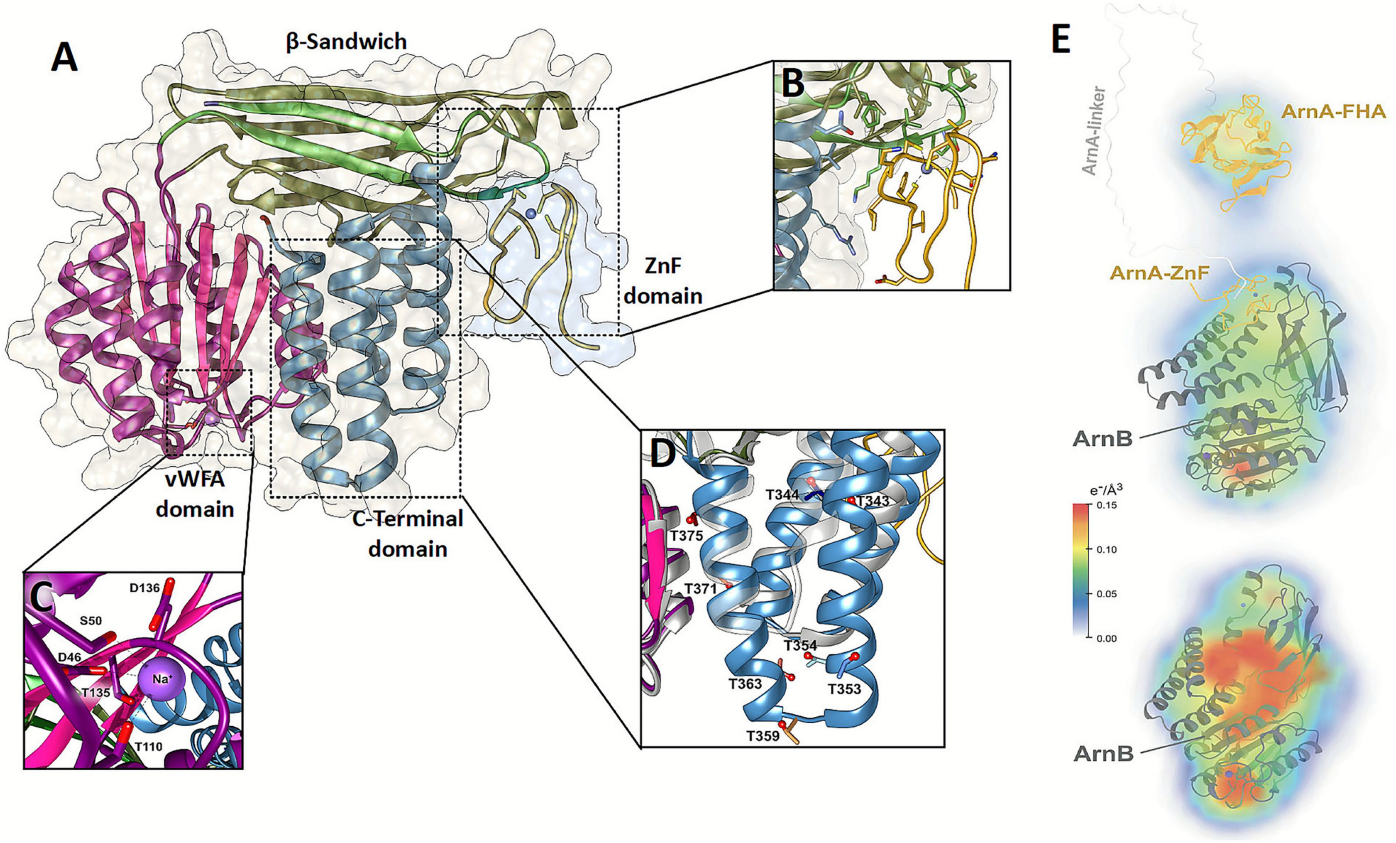
Overall structure of the ArnAB complex and comparison with ArnE. **(A)** Full structure of ArnAB complex and domain overview showing the ArnA ZnF domain interaction site. Domains are color-coded as follows: β-sandwich (green), vWA (pink/purple), C-terminal domain (blue), ZnF domain (yellow). **(B)** Focus view on the ZnF interaction site with important residues showing as stick models. **(C)** MIDAS site of the vWA domain of ArnB reveals Na^+^ interacting residues. **(D)** ArnB and ArnE (vWA2) (grey) superposition with secondary structure elements numbering. The magnification shows the C-terminal domain of ArnB with threonine residues shown as sticks and OH-groups as balls, structurally highlighting potential FHA interaction sites. **(E)** *Ab initio* envelope as calculated from SAXS-derived scattering vectors using 20 iterations of DENSS for electron density modeling and ScÅtter for refining GNOM-processed SAXS data. For the ArnAB complex (top) a *D*_max_ value of 15.7 nm was derived (*R*_g_ 4.16 nm), the DENSS model density has a resolution of 33 Å; the values for ArnB (bottom) alone are *D*_max_ = 7.5 nm, *R*_g_ = 2.41 nm, resolution = 30 Å. Structural models for the FHA domain of ArnA (orange), ArnB (grey) and the ArnB/ArnA-ZnF complex were fitted by ChimeraX into SAXS densities.

During efforts to obtain crystals of the phosphorylated ArnAB complex, which were fruitless, we were surprisingly able to determine the structure of unphosphorylated ArnB (UniProt: Q4J9H3, Saci_1211) complexed to ArnA (UniProt: Q4J9H4, Saci_1210) at 2.1 Å resolution. Cocrystals of unphosphorylated ArnAB comprise two complexes per asymmetric unit, whose structures were solved by molecular replacement. The ArnAB complexes are defined by electron density for residues T2-S380 of ArnB and the N-terminal zinc-finger (ZnF) domain of ArnA (P16-K42, Figures 1A,1B). The latter implies loss of the FHA domain and the linker region of ArnA during crystallization, possibly by unspecific proteolysis as observed before when solving the structure of the C-terminal ArnA FHA domain (Hoffmann et al., 2019). The overall architecture of ArnB closely resembles its paralog ArnE, including the vWA domain, an eight-stranded β-sandwich (β1β2β9-β14) whose topology is split by the vWA domain, and the C-terminal RHB domain (*α*6-α9), that has been classified before as ArnB_C domain (InterPro entry IPR040929) (Figure 1A) (Hoffmann et al., 2016, 2019). Unlike ArnE, the RHB region of ArnB harbors an elongated helix pair α8-α9 in the second half of the motif, revealing one of the most substantial structural differences between ArnB and its paralog ArnE. This extension has multiple threonine residues that could promote potential interaction with the FHA domain of ArnA like T343/T353 in the phosphorylated state (Figure 1D). Compared to ArnE, ArnB features a short helical segment (Figure 1A, α’, P95-Q103) in the vWA domain between β5 and α2 (for topology assignments see (Hoffmann et al., 2019)). The vWA domain of ArnB harbors like ArnE a sodium ion coordinated by D46, S50, T110, T135 and D136 in the metal ion-dependent adhesion site (MIDAS, Figure 1C).

While ArnA interacts strongly via its FHA domain to phosphorylated ArnB (Hoffmann et al., 2019), the ArnAB complex in its unphosphorylated state shows that the zinc finger (ZnF) domain alone is sufficient to promote interaction. This ZnF domain belongs to the RanBP2-type (IPR001876) and is characterized as a ZnF ribbon domain by two consecutive, distorted β-hairpin motifs, which coordinate together a zinc ion via C21, C24, C35 and C38. The interface of the ZnF domain of ArnA with ArnB has a rather moderate size of 504/574 Å^2^ for ArnAB chains A/C and B/D, respectively. The interactions are mostly of hydrophobic nature and include the C-terminal β-hairpin motif (D31-Q41) of the ZnF domain as well as the N-terminal β1-β2 loop (H12-K21) of the β-sandwich domain, its α5 linker to the RHB domain (V286-I293) and adjacent residues of the RHB domain facing the ZnF domain (Figure 1B). Accordingly, the ArnA-ArnB interaction based on the ZnF domain appears to be rather weak as indicated by pulldown assays (Supplementary Figure S1A), but traceable by mass photometry (Ye et al., 2020). A further point for the uniqueness of the ZnF-mediated ArnA-ArnB interactions are AlphaFold2 models, which were unbiased of the ArnAB structure and show almost an identical interaction as in the ArnA-ArnB cocrystal structure for 4 of the 5 predicted ArnA–ArnB models (Supplementary Figure S2) with displacement r.m.s.d. values of 1.57–1.65 Å for M1-Q25 of the ZnF domain relative to the ArnB domain. This indicates that the intrinsic sequence covariation for ArnA and ArnB domains is already sufficient to provide a robust predictor for the relatively small ArnB/ZnF domain interface. Moreover, an ArnA–ArnB interaction is also displayed in solution by SAXS data (Figure 1E) as the *ab initio* envelope as derived from the SAXS data is fittable to the ArnA-FHA and ArnAB crystal structures. Here, the pair distance distribution function *P*(*r*) suggests an overall elongated shape (Supplementary Figure S3) and thereby supports an ArnA-ArnB interaction based on the ZnF domain, as in the crystal structure, with a flexible region followed by an unbound FHA domain due to a lack of pThr anchor sites.

Overall, structural analysis of the unphosphorylated ArnAB complex reveals key differences between ArnB and its apparently non-functional paralog ArnE, particularly in the RHB region, and an alternative binding mode of ArnB for ArnA.

### 2.2 Promiscuity of ArnB’s phosphorylation-dependent interaction sites for ArnA

**Figure 2:**
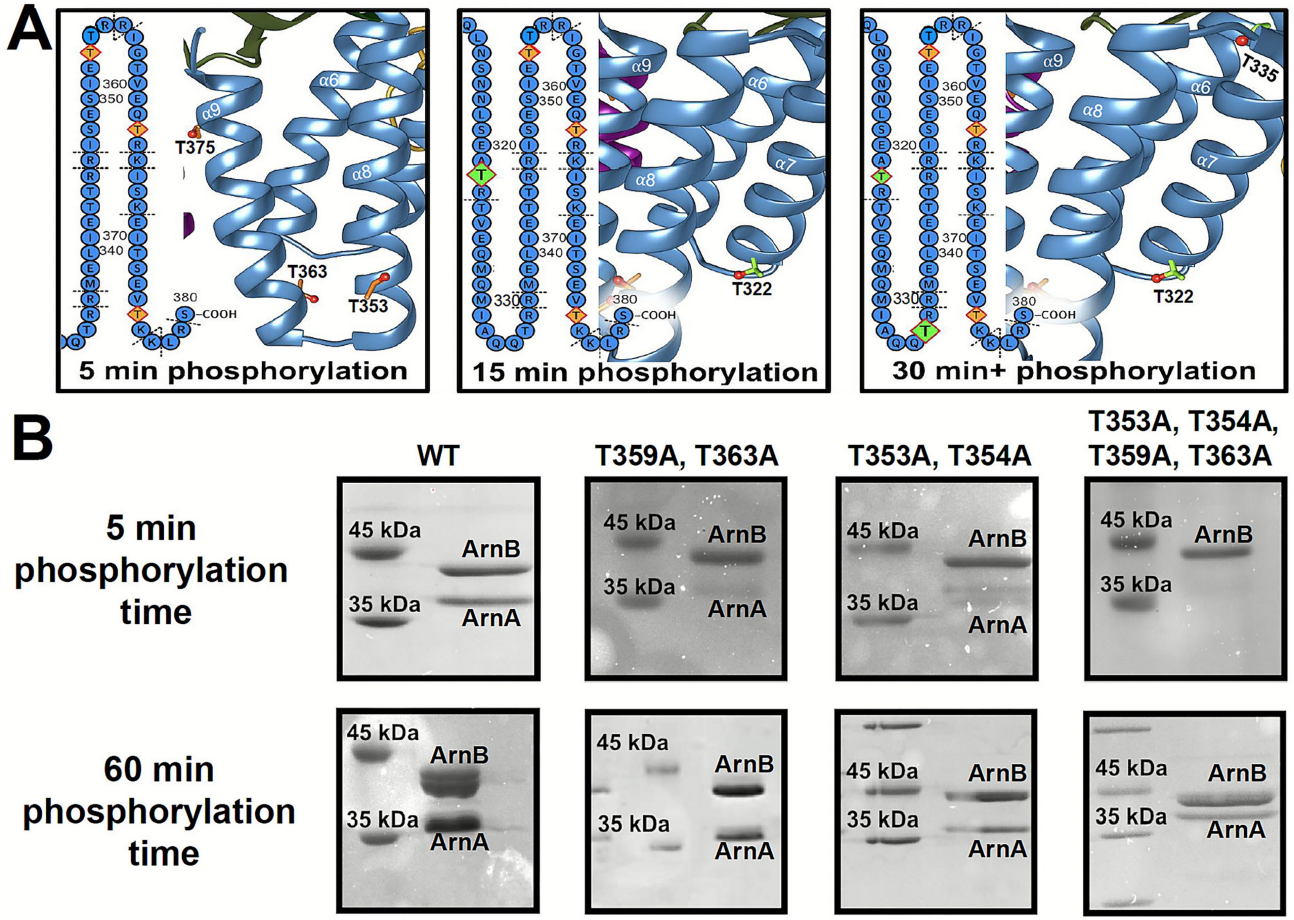
Promiscuity of ArnB phosphorylation sites. **(A)** Impact of phosphorylation time on threonine phosphorylation by MS. Orange squares mark pThrs after 5 min of incubation with ArnC; green squares indicate pThrs formed later. **(B)** SDS PAGEs of gel-pulldown assays showing that phosphorylation time has an impact on ArnB mutants. Several ArnB mutants (upper bands) are not able to interact efficiently with ArnA (lower bands) at short phosphorylation times, causing a loss of the ArnA band.

To identify the interaction site of ArnB in the phosphorylated ArnAB complex, we performed a comprehensive mass spectrometry-based analysis. Compared to ArnE, our structural analysis of ArnB showed major differences for the RHB motif thus making it the most likely interaction site. We therefore examined the phosphorylation pattern by the Hanks-type kinase ArnC (UniProt: Q4J9J0, Saci_1193) under previously reported phosphorylation conditions (Ye et al., 2020). Accordingly, we observed phosphorylation of many threonines within the RHB motif during *in vitro* phosphorylation experiments (Figure 2, Supplementary Dataset 5), raising questions about the native phosphorylation conditions.

To pinpoint these phosphorylation sites, we analyzed the effect of different incubation times on ArnB’s phosphorylation pattern using ESI-MS of tryptic fragments. We tested time points of 5, 15, 30, and 60 min, with 60 min leaving the phosphorylation pattern after 30 min unaffected (Supplementary Dataset 5). Three threonines, T353, T363, and T375, in the elongated helix pair α8-α9 of the RHB were phosphorylated almost to completion within the first 5 min (Figure 2A, Supplementary Dataset 5), supporting the notion that phosphorylation often occurs in clusters (Nishi et al., 2014). T353 is part of the SIETT motif (S350-T354) that appears duplicated in ArnB from the LIETT motif (L340-T344) at the N-terminus of helix α8, which undergoes no phosphorylation despite surface exposure of the corresponding residue T343. In ArnE, two of these threonines, T353 and T363, are lacking, the former one due to an absence of the SIETT motif. Accordingly, ArnE was found not to interact with ArnA (Reimann et al., 2012). In contrast, two other pThr sites, T322 and T335, were found to be phosphorylated only after 15 min in the α7-α8 pair of the RHB. Notably, these sites are also shared by ArnE. Overall, multiple phosphorylation of the extended α8-α9 pair of the RHB domain primes this region for interaction with the FHA domain of ArnA.

As predicted, ArnA could be efficiently pulled down by tagged ArnB already after 5 min of phosphorylation (Figure 2B). To narrow down direct pThr sites for FHA domain interaction, we performed alanine mutagenesis on crucial motifs. We substituted key threonines to alanine, specifically T353A/T354A in the SIETT motif and the T359A/T363A pair. Both substitution pairs diminished the FHA domain from interacting with ArnB when phosphorylation was limited to 5 min or less (Figure 2B). As reported before with long phosphorylation times (Hoffmann et al., 2019), increasing the phosphorylation time to 60 min allowed ArnA to be successfully pulled down with ArnB again, indicating that prolonged off-target phosphorylation compensates for these substitutions. Such an off-target interaction was also observed for a mutant with multiple T→A mutations (T343A/T344A/T353A/T354A/T359A/T363A/T371A/T375A) in the α8-α9 pair of the RHB (Supplementary Figure S1B). Overall, the FHA domain interacts specifically with the pThr sites of the α8-α9 pair, while phosphorylation in the remainder of the ArnB domain allows lesser specific ArnA-ArnB interaction.

The observed hyper-phosphorylation in the RHB domain may cause structural rearrangements, which may not only provide direct interaction sites but unmask pThr sites for a proper ArnB-FHA interaction.

### 2.3 HDX-MS reveals phosphorylation-dependent ArnB rearrangement primed for ArnA interaction

To explore the interaction site(s) between ArnA and phosphorylated ArnB in solution, we utilized hydrogen/deuterium exchange mass spectrometry (HDX-MS). This technique makes use of the property of amide protons to exchange deuterium from the solvent, allowing us to trace conformational changes and interaction interfaces. We subjected ArnA, ArnB, phosphorylated ArnB, and the ArnA/phosphorylated ArnB complex to HDX-MS experiments, focusing on (i) the conformational changes of ArnB upon phosphorylation, and (ii) the interaction regions between ArnA and phosphorylated ArnB.

In our analysis we identified 80 peptides for ArnA and 145 for ArnB, that covered more than 90% of their corresponding amino acid sequences (Supplementary Figures S4A, S5A, Dataset 1). The HDX profile of ArnA corroborates its predicted domain topology, in particular highlighting the disordered nature of the linker (S28-N99) that connects the ZnF and FHA domains, which showed maximal HDX after 10 s after deuteration (Supplementary Figure S4B).

**Figure 3:**
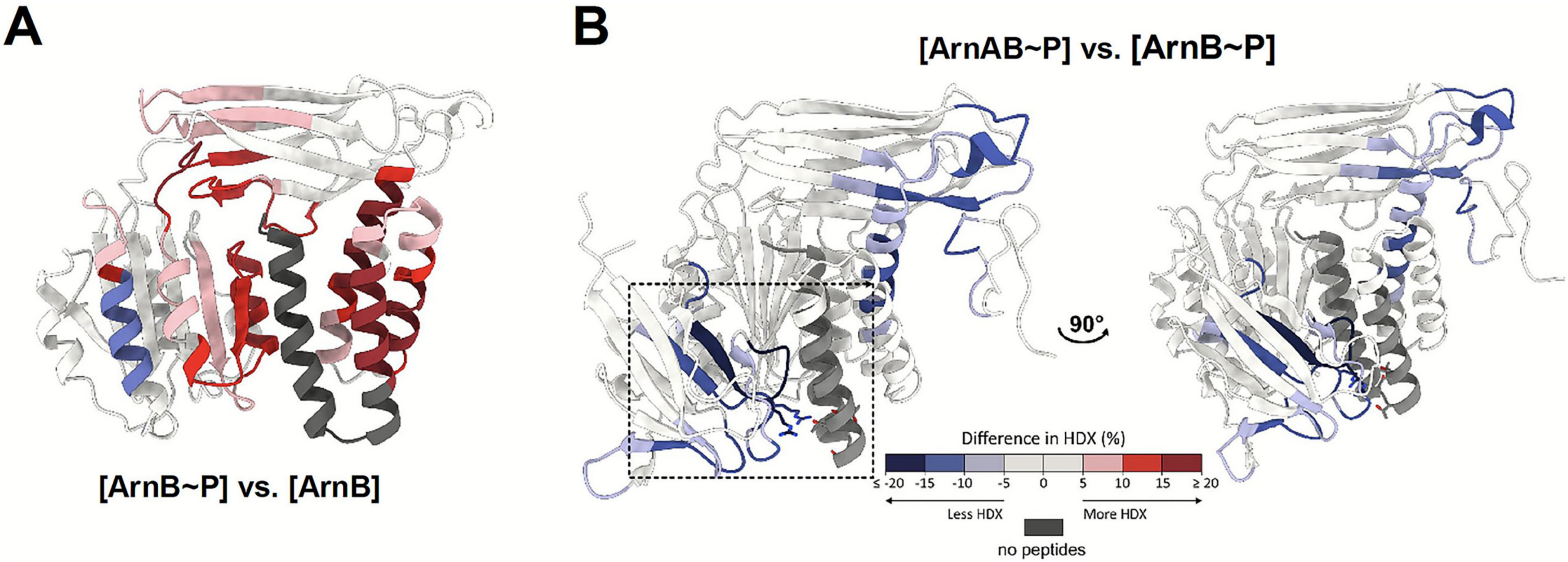
HDX-MS reveals impact of RHB phosphorylation on ArnB and its ArnA binding. **(A)** Impact of phosphorylation of ArnB (ArnB~P) vs. unphosphorylated ArnB as shown by cumulative HDX-MS, i.e., highest scoring change per given time point (Supplementary Figure S6). The changes in HDX suggest a structure-wide impact of RHB phosphorylation. **(B)** Cumulative HDX of the phosphorylated ArnAB (ArnAB~P) complex vs. phosphorylated ArnB. The dashed box focuses on the FHA interaction site at the RHB as possible interaction site. The legend is representative of the whole figure.

Phosphorylation of ArnB alone already induces widespread conformational changes compared to its unphosphorylated state, particularly for the whole RHB as far as covered by HDX-MS (α6-α8, L318-E352), which exhibits the strongest HDX increase, parts of the vWA domain contacting the RHB (α3-β8) as well as the linker between the vWA and β-sandwich domains including helix α4 (Figures 1A, 3A, Supplementary Figures S6A,B). Furthermore, regions of the β-sandwich contacting the RHB and vWA domain exhibit increased disorder (L222-V238, β10-β11) suggesting partial unfolding or disentanglement of the three-domain assembly upon RHB phosphorylation. Interestingly, ordering of the linker between the β-sandwich and the RHB is also apparent, but to a lesser degree than in the ArnAB complex.

Upon ArnAB complex formation with phosphorylated ArnB, major HDX reduction became apparent for the FHA domain, being the major interaction site for pThr sites, and to a lesser extent for the ZnF domain (Supplementary Figures S4C,D). The phosphate recognition module of the FHA domain constituted by R132 and R147 exhibited the strongest HDX reduction, underscoring its role in strengthening the ArnA/ArnB interaction (Hoffmann et al., 2019). For phosphorylated ArnB, the HDX profile revealed regions of higher-order upon complexation with ArnA (Supplementary Figure S5B), i.e., lower HDX at 10 s of deuteration and progression in HDX over the time-course (Figure 3B, Supplementary Figures S6C, S7). This ordering relative to phosphorylated ArnB involves the linker region between the β-sandwich and the RHB, including helix α5 and the beginning of α6 of the RHB. Furthermore, binding of ArnA to phosphorylated ArnB caused reduction of HDX rates near the binding site of the ArnA-ZnF domain implying some conformational change in this region (Figure 3B, Supplementary Figures S6B,C). Helix α6 in the RHB of ArnB (D295-A310) incorporated less deuterium than in the phosphorylated state of ArnB alone (Supplementary Figure S6) suggesting that phosphorylation-induced conformational changes may facilitate tight ArnA binding.

Overall, our HDX-MS analysis is consistent with the notion that ArnB phosphorylation in the RHB results in partial disintegration or dynamic loosening of its intimate three-domain arrangement and weakens thereby the interaction site with the ArnA-ZnF domain (Figure 1). Instead, this disordering exposes the pThr sites of the RHB’s α8-α9 helices for interaction with the ArnA-FHA phosphate recognition module and possibly for other downstream signaling components.

### 2.4 tims-ToF proteomics analysis of arnA and arnB *S. acidocaldarius* deletion strains

**Figure 4:**
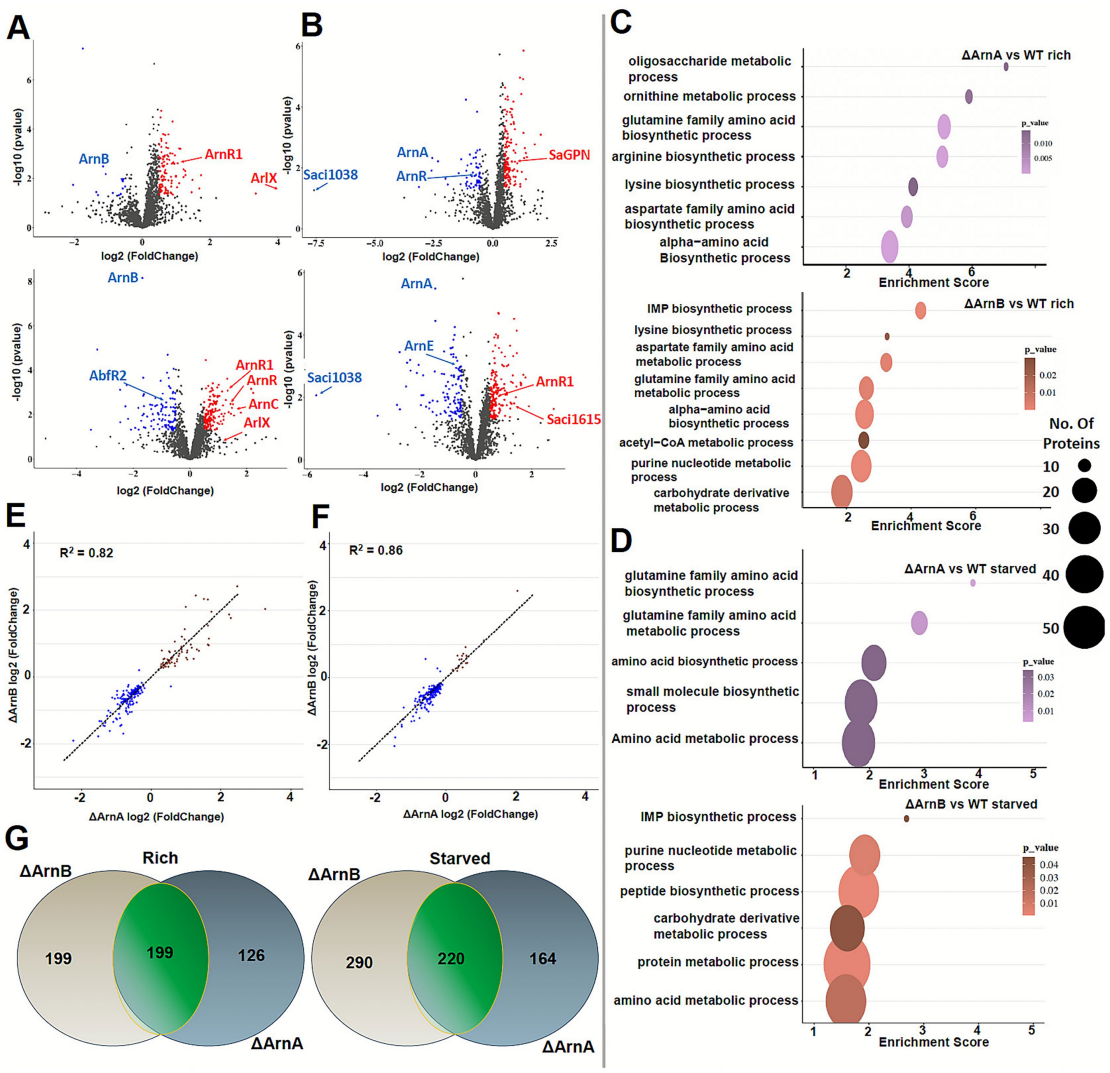
Proteomics analysis of *Sulfolobus acidocaldarius* Δ*arnA* and Δ*arnB* strains in nutrient-rich and starved conditions compared to the wild type. **(A,B)** ArnA/B knockout proteomic analysis visualized as volcano plots in respect to the corresponding WT nutritional state. The top plots show nutrient rich conditions and the bottom plots nutrient starved conditions. The sample size corresponds to biological triplicates per variant, including respective WT samples, per measurement two technical duplicates were made. Protein levels that have *p*-value <0.05 and a log_2_ fold change of >0.5 (i.e., >50%) are colored blue, i.e., down regulated in the respective knockout and nutritional state, or red, i.e., up regulated in the respective knockout and nutritional state. **(C,D)** Gene enrichment analysis of respective protein hits, clustering them into GO terms revealing mostly metabolic involvement. **(E,F)** Scatter plots of proteins found in both ArnA and ArnB knockout reveal high cross-effected protein rate, highlighting their cooperative role in *S. acidocaldarius*. **(G)** Venn diagrams of ArnA and ArnB knockouts show that only ~half of the affected proteins (*p* < 0.05) can be found in both knockouts, leaving room for individual roles.

To uncover the *in vivo* regulatory impact of ArnA and ArnB under nutrient limitation, we compared the proteomes of *S. acidocaldarius* Δ*arnA,* Δ*arnB* and Δ*arnE* strains to the wild type under nutrient-rich and starved conditions. Lysates were analyzed using a timsTOF mass spectrometer with label-free quantification. Volcano plots were generated to visualize proteomic shifts (Figures 4A,B, Supplementary Figures S8, S9, S11), indicating major impacts on the proteomic profiles due to deletion of arn components. Our analyses identified 1,699–1,710 proteins per sample leading to the identification of 1,723 overall proteins of the 2,222 known gene products for the respective *S. acidocaldarius* strain. The deletion strains showed 1,713–1,716 proteins with 98.7–99.4% overlap.

Compared to the wildtype proteome, a two-tailed t-test with a *p*-value cutoff of <0.05 revealed significant changes for 325 (rich) and 384 (starved) gene products in the Δ*arnA* strain and 398 (rich) and 510 (starved) in the Δ*arnB* strain, respectively. These proteomic changes by *arnA* and *arnB* knockouts are comparably minor as the Δ*gpn* strain of the GPN-loop GTPase *Sa*GPN (Korf et al., 2023) caused global changes in the *S. acidocaldarius* proteome (*p* < 0.05: 773 for rich; 753 for starved condition). In addition, we applied a log2-fold change threshold of 0.5 for accounting abundance changes of biological relevance (Figures 4A, 4B), yielding 115 (rich)/243 (starved) gene products for the Δ*arnA* and 185 (rich)/279 (starved) gene products for the Δ*arnB* strain. These results indicate that the proteomic impact of the Δ*arnA* and Δ*arnB* knockouts is more pronounced under nutrient-starved conditions. In contrast, the Δ*arnE* strain, which shows no motility phenotype (Hoffmann et al., 2019), revealed surprisingly a significantly higher number of affected gene products, with the given filtering yielding 351 (rich) and 390 (starved) hits, but no direct effect of the nutrient conditions.

Next, we conducted gene ontology (GO) enrichment analysis on the proteomic changes (Figures 4C,D) that may reflect biological effects of the *arnA* or *arnA* deletions in *S. acidocaldarius*. This analysis revealed that both knockouts are enriched for metabolic enzymes, especially those involved in nitrogen-containing amino acid pathways. Additionally, enzymes linked to purine/nucleotide metabolism, acetyl-CoA metabolism and carbohydrate metabolism were significantly enriched in both Δ*arnA* and Δ*arnB*. Notably, when comparing the significantly altered proteins between the two deletion strains, only overlaps were observed for 199 (rich) and 220 (starved) affected gene products (Figure 4G). However, within this overlap, the correlation in fold change of affected gene products was high, i.e., 82% (rich, Figure 4E) and 86% (starved, Figure 4F). This suggests that around half of the observed proteomic changes in each knockout derived from the shared regulatory role of ArnA or ArnB, presumably via the physical interaction as shown above*. Vice versa,* the remaining changes appear to be specific for each regulator, indicating functions of ArnA and ArnB that are at least partially independent of each other. For example, in the Δ*arnB* strain, the largest abundance change (>30 fold) has been observed for Saci_1,038, a gene product (Uniprot: Q4J9Y4) related to a membrane-tethered solute binding protein (Figure 4B).

Interestingly, in Δ*arnA*, the ArnB levels are reduced compared to the WT, and ArnA levels in Δ*arnB* are similarly diminished (Figure 4A), emphasizing the importance of complex formation on either the stability or expression of ArnA and ArnB. In addition to ArnA and ArnB, other proteins of the archaellum regulatory network were identified, including the Hanks-type kinase ArnC. We previously observed that upon starvation, both the transcript and protein levels of ArnC increase, and its deletion leads to reduced motility (Hoffmann et al., 2016). Interestingly, here we found that ArnC protein levels are even more elevated upon starvation in Δ*arnA* and Δ*arnB* compared to the WT, suggesting a functionally linked role in the ArnA/ArnB-dependent regulation of motility.

Several putative transcription regulators responded to starvation, suggesting secondary regulatory effects downstream of nutrient sensing. Among the downregulated proteins upon starvation was Saci_1223, a Lrs14-like positive biofilm regulator (AbfR2) (Recalde et al., 2021), and Saci_1615, a ZnF_C2H2-domain protein, which showed increased abundance in the *ΔarnA* and *ΔarnB* strains compared to the wildtype. Notably, the biofilm regulator AbfR2 was elevated in the Δ*arnA* background. A comparison of these proteomics profiles with the previously studied Δ*gpn* strain, lacking the GPN-loop GTPase *Sa*GPN (Saci_1281) and exhibiting diminished motility, (Korf et al., 2023) shows that the proteome changes of the hypermotile Δ*arnA* and/or Δ*arnB* variants (Reimann et al., 2013) are modest. This suggests that ArnA and ArnB act downstream in the hierarchy of the archaellum regulatory network, fulfilling a more specific influence on motility-related processes. In contrast, *Sa*GPN appears to function higher up in the network, affecting a broader cellular regulation due to its large-scale proteomic changes upon deletion. Accordingly, no significant correlation was found between differentially abundant proteins in the Δ*gpn* strain and those in either the *arnA* or *arnB* deletion strains (Supplementary Figure S10). This suggests that ArnA and ArnB operate independently of *Sa*GPN and likely subordinate to *Sa*GPN within arn.

Surprisingly, the analysis for ArnE revealed both parallels and divergences from its paralog ArnB. While Δ*arnE* and Δ*arnB* show some overlapping changes for the gene ontologies, particularly in nucleotide metabolism and IMP biosynthesis as well as carbohydrate derivative metabolism, the Δ*arnE* strain uniquely impacts iron–sulfur cluster assembly, ATP-binding, and rRNA proteins and exhibits stronger regulation of amino acid biosynthetic pathways (Supplementary Figures S11A,B). However, the proteomic changes of the *ΔarnE* strain lack any correlation to the changes in Δ*arnA* and Δ*arnB* strains (Supplementary Figures S11C,D), thereby setting apparently the biological function of ArnE aside to the ArnAB complex.

## 3 Discussion

**Figure 5:**
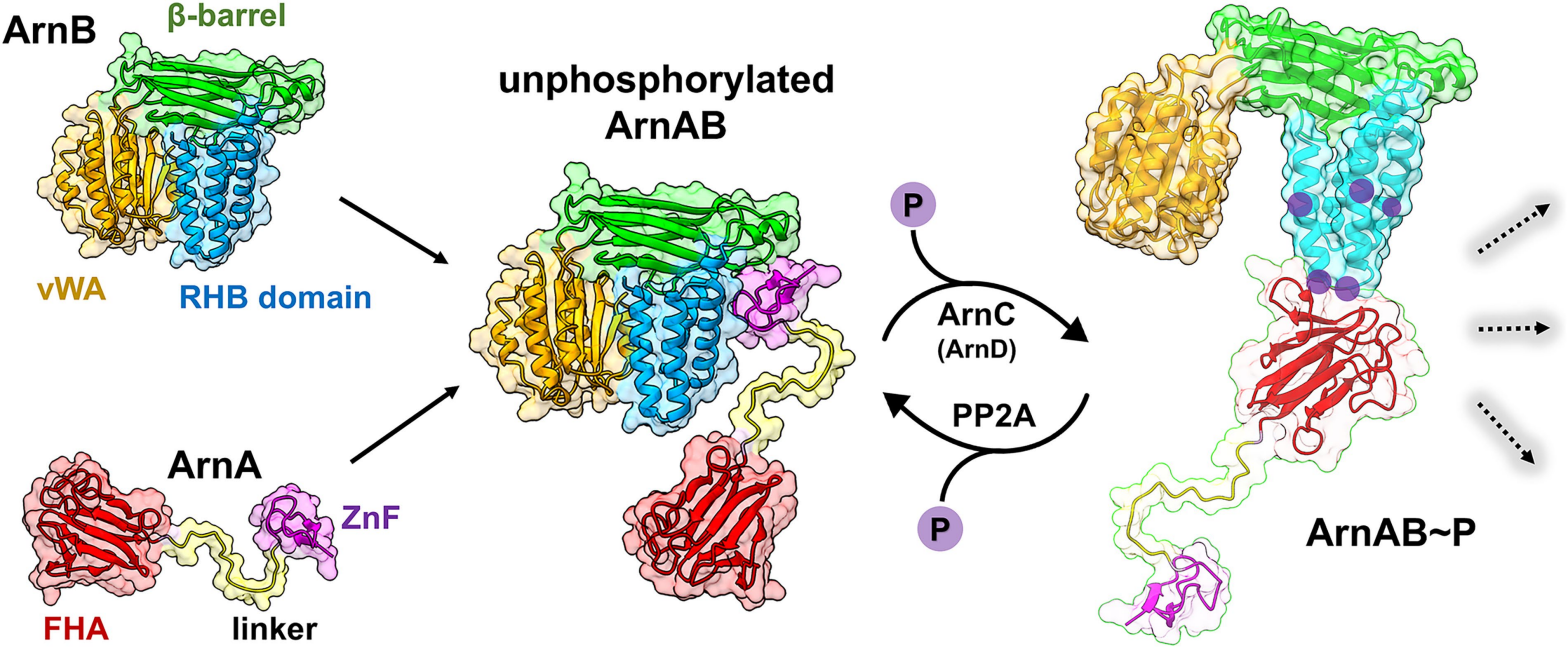
Phosphorylation-dependent conformation of the ArnAB complex. Phosphorylation of ArnB causes switching of a loosely bound, unphosphorylated ArnAB complex by tethering to the ZnF domains to a tightly bound complex by FHA-pThr interaction. This switching plays a role in the archaellum regulatory network and several metabolic pathways. Masking/unmasking of the ZnF domain and regions of the tripartite ArnB domain may allow further downstream interactions for signaling.

This study provides the first high-resolution structural and dynamic view of the unphosphorylated ArnAB complex, advancing our understanding of motility regulation in *Sulfolobus acidocaldarius*. While ArnA and ArnB have long been implicated in the archaellum regulatory network (Hoffmann et al., 2019; Ye et al., 2020), the mechanism of phosphorylation-dependent signaling remained unclear. Our combined structural, biochemical, and proteomic analyses show that the ArnAB complex acts as a phosphorylation-sensitive molecular switch, linking nutrient availability to motility via a dynamic shift between a loose, unphosphorylated and a tight, phosphorylated complex (Figure 5).

The structure of unphosphorylated ArnAB reveals that ArnA interacts with ArnB via its ZnF domain, while HDX-MS identified a second interaction interface involving the phosphorylated RHB of ArnB and the FHA domain of ArnA. Interestingly, ArnB undergoes sequential and site-specific phosphorylation by ArnC, beginning with a pair of surface-exposed threonines located in the tip regions of the elongated helix pair α8-α9 of RHB (T353-T363) and progressing toward residues buried in the interface between the RHB and the vWA domain, T371-T375. This stepwise phosphorylation leads to conformational remodeling of the ArnAB complex, exposing previously inaccessible sites by high-affinity binding to ArnA’s FHA domain (Figure 5). Such regulated flexibility is similar to other systems where phosphorylation triggers order-to-disorder transitions (Querfurth et al., 2011; Kumar et al., 2018). For example, in the ankyrin-repeat protein p19^INK4d^, sequential phosphorylation within the helical repeat domain progressively destabilizes the N-terminal half of the protein, ultimately leading to the unfolding of that section (Kumar et al., 2018). Moreover, the casein kinase 1a (CK1a) in *Neurospora* progressively hyper-phosphorylates the clock protein FREQUENCY (FRQ), triggering a conformational change driven by clustered phosphorylation (Querfurth et al., 2011). Another example for protein–protein interaction triggered by hyperphosphorylation is the cyclin–Cdk1–Cks1 system (Kõivomägi et al., 2013). Our mutational analysis revealed that no single phosphorylation site is essential, as long as alternative pThr sites are available in the RHB, supporting a redundant and adaptable interaction mechanism. This redundancy may allow *S. acidocaldarius* to fine-tune motility responses by different phosphorylation patterns of the RHB.

Intriguingly, we found that the structural characteristics of ArnB with its three-domain architecture is a structural blueprint widely distributed among all domains of life, although mostly not being involved in a multi-phosphorylation dependent processes like in ArnAB. In other prokaryotes, more structural orthologs with ArnB architecture can be found like vWA from the actinobacterium *Catenulispora acidiphila* (Hoffmann et al., 2019) or the predicted structure of YfbK from *E. coli*. (Supplementary Figure S12A). In archaea, structurally related gene products are found in Euryarchaeota like *Halorubrum* or Korarchaeota and Heimdallarchaeota (Supplementary Figure S12B). In eukaryotes, the homologous Sec23 and Sec24 factors of the COPII vesicles have an ArnB-like domain arrangement as their core motifs consist of a vWA and β-sandwich domain that is followed by a helical domain similar to RHB (Supplementary Figure S13). Like ArnA, Sec23/Sec24 even includes an N-terminal ZnF domain besides a gelsolin-type C-terminal domain. Although ArnB lacks this C-terminal domain of Sec23/24, the FHA domain of ArnA is predicted to interact in a phosphorylation-dependent manner with the RHB that resembles the packing of the Sec23/Sec24 motif and the gelsolin domain. Surprisingly, the closest structural hits for the ArnB architecture outside the archaeal domain are found in the plant and fungal kingdoms for WAV3 E3 ubiquitin ligases despite marginal sequence conservation (Supplementary Figure S12C).

Despite prior suggestions of a transcription factor role for ArnA in *S. islandicus* (Jiang et al., 2023), we found no hint that ArnA and ArnB binds directly DNA. This supports a regulatory function that is likely independent of direct DNA interaction, potentially mediated through other DNA-binding proteins. First, there are no structural indications of DNA binding sites in either ArnA or ArnB. Secondly, in the unphosphorylated state, the zinc finger domain of ArnA is unavailable for DNA binding as it forms interactions with ArnB. However, in the phosphorylated state the ZnF motif can be predicted to get released due to conformational changes of its ArnB interaction site and the recruitment of the ArnA FHA domain to the RHB. As recombinant *Sis*ArnB was also purified from a homologous host, *S. solfataricus*, its reported DNA binding in the presence of *Sis*ArnA activity may depend on additional factors, otherwise lacking in recombinant proteins from *E. coli*. One of them is ArnB phosphorylation with the triggering of structural changes and tight ArnAB association, others may be down-stream DNA-binding factors recognized by the phosphorylated ArnAB complex.

Proteomic profiling of *ΔarnA* and *ΔarnB* revealed that ArnA and ArnB not only regulate archaellum expression but also affect metabolic pathways. The enrichment of enzymes involved in nitrogen-rich amino acid synthesis, purine/nucleotide metabolism, carbohydrate and acetyl-CoA metabolism point to broader roles in balancing motility with energy status. These findings are consistent with the transition from a sessile biofilm-forming state to a motile planktonic lifestyle. Besides metabolic proteins, transcriptional and regulatory proteins were also found to be regulated upon starvation, including biofilm transcriptional regulator AbfR2, hinting at their possible influence on archaellum expression and the switch between biofilm production (with nutrient access) and motility (nutrient starvation). When compared to the *Δgpn* strain, which exhibits broad proteomic disruption and reduced motility due to a lack of the GTPase *Sa*GPN (Korf et al., 2023), the changes in *ΔarnA* and *ΔarnB* strains are more focused, suggesting that ArnA and ArnB operate downstream of *Sa*GPN within the hierarchy of the archaellum regulatory network. Their narrower impact supports the model of ArnAB as a modularity hub, integrating phosphorylation signals into motility control without globally rewiring the proteome. Notably, considering *Sa*GPN as part of the arn system, a physical association of the ArnAB complex with *Sa*GPN may suggest a potential collaborative function of all three components. This could explain why the previously reported PP2A co-IP assay (Ye et al., 2020) pulls down not only ArnA and ArnB but also *Sa*GPN. However, this finding does not necessarily suggest a direct physical interaction of the ArnAB complex with *Sa*GPN.

The co-regulation of ArnA and ArnB protein levels, each being reduced in the absence of the other, supports a mutual stabilization mechanism dependent on complex formation. This, combined with the increased ArnC levels observed in both knockouts, points to a feedback regulation within the arn system, ensuring a rapid response to nutrient stress. Our integrative analysis suggests that ArnA and ArnB form a phosphorylation-tuned regulatory module whose structural dynamics and oligomerization state modulate archaellum expression in response to nutrient availability. This regulation is largely influenced by the phosphorylation state of ArnB, regulated by the PP2A/ArnC phosphatase-kinase system. While the stand-alone domain of PP2A lacks complex regulation, ArnC provides an array for potential regulatory inputs, thanks to its extended N-terminal region with an OB domain followed by a long tetratricopeptide-like stretch and the C-terminal Ser/Thr kinase domain. As transcript and protein levels of ArnC are elevated during starvation and cell division (Lundgren and Bernander, 2007; Jarrell et al., 2021), its specific activity on the RHB region of ArnB may further boost the action of the constitutively active *S. acidocaldarius* kinase ArnD (Hoffmann et al., 2016) in the presence of its counterplayer, the PP2A phosphatase.

Overall, the phosphorylation-dependent ArnAB module emphasizes the complexity and versatility of the arn system in modulating protein interactions and functions. For comparison, the ArnB paralog ArnE with its lack of phosphorylation sites in the C-terminal *α*-helical domain has a clearly distinct role, showing only minimal overlap with the motility-related changes characteristic of Δ*arnA*/Δ*arnB* strains. Under starvation, ArnE’s regulatory network expands to include phospho-dependent metabolic processes as well as hydrolase and transferase activities, responses not seen in ArnB knockouts. Likewise, changes for proteins harboring iron–sulfur cluster may further underscore a specialization of ArnE as a metabolic sensor, operating alongside but distinctly from the ArnAB complex’s phosphorylation-dependent motility control. These findings reveal how gene duplication and divergence created complementary regulatory systems, where ArnAB affects primarily motility regulation, whereas ArnE affects metabolic regulation.

## 4 Methods

### 4.1 Materials

If not stated otherwise chemicals and materials were obtained from Carl Roth. Primers were ordered from Microsynth.

### 4.2 Recombinant overproduction of ArnA, ArnB, and ArnC

Recombinant proteins (ArnA: Saci_1210, ArnB: Saci_1211, ArnC: Saci_1212: Saci_1193) were expressed from plasmids pSVA1036-ArnA-N strep and pSVA1036-saci1211 using the pETDuet-1 vector backbone (Supplementary Data). containing a C-terminal 6xHis-tag, under control of the *lac* promoter, and carrying an ampicillin or kanamycin resistance gene. Overproduction of recombinant proteins for this study was performed in LB medium (10 g/L tryptone, 10 g/L NaCl, 5 g/L yeast extract) supplemented with 34 μg/mL chloramphenicol, using BL21(DE3) Rosetta cells. After induction with 0.5 mM IPTG at OD_600_ of 0.6–0.8, cultures were incubated for 18 h at 16 °C and 150 rpm. Harvesting (20 °C, 5,422 x *g*, 20 min) was followed by resuspension in lysis buffer (150 mM NaCl, 50 mM Tris, pH 8.0). Cell pellets were either used freshly for protein purification or frozen in liquid N_2_ and stored at −80 °C for later use.

### 4.3 Purification of unphosphorylated ArnA and ArnB

Cell pellets were lysed using a micro-fluidizer (2 min/2 L cell pellet equivalent) after harvesting. The lysate was centrifuged (39,000 x *g*, 20 °C, 20 min), the supernatant heat-treated (70 °C, 20 min) and centrifuged again (39,000 x *g*, 20 °C, 20 min) before being loaded to a Ni-NTA column (5 mL, Cytiva). After sample application, the column was washed with 9 column volumes wash buffer (150 mM NaCl, 50 mM Tris, 25 mM imidazole, pH = 8.0) and eluted with 5 column volumes elution buffer (150 mM NaCl, 50 mM Tris, 500 mM imidazole, pH = 8.0). The elution was concentrated (<2.5 mL) and applied to a HiLoad® 16/60 Superdex® 200 pg. (Cytiva) for size-exclusion chromatography (SEC), which had been equilibrated with running buffer (150 mM NaCl, 50 mM Tris, pH = 8.0). Fractions containing the proteins, validated by SDS-PAGE, were collected, concentrated, frozen in liquid N_2_ and stored at −80 °C.

### 4.4 Phosphorylation-dependent pull down of the ArnAB complex

To generate phosphorylated ArnA–ArnB complexes, we used a pre-lysis phosphorylation approach in which ArnC was supplied post-expression but before cell disruption. *arnA* and *arnB* genes were co-expressed in *E. coli* from the plasmid encoding His₆-tagged ArnB and StrepII-tagged ArnA (Supplementary Figure S14), while ArnC was overproduced separately as untagged protein using a modified variant of the pSVA1009 vector (Supplementary Figure S16). After harvesting, the ArnC cell pellet from 1 L culture was combined directly with the pellet from a 1 L ArnA/ArnB culture, and the mixed material was resuspended together in lysis buffer (50 mM Tris pH 7.5, 150 mM NaCl, 10 mM imidazole, protease inhibitors). This setup ensured that ArnC was present during and immediately after cell lysis, allowing phosphorylation of ArnB prior to pull down by affinity purification. Cells were lysed using a micro-fluidizer, the lysate was clarified, and ATP and MnCl₂ were added to final concentrations of 2.5 mM each before incubation at 55 °C for defined times (5–60 min), enabling controlled ArnC-mediated phosphorylation. Co-elution of ArnA with His-ArnB from Ni–NTA affinity columns after phosphorylation reflects phosphorylation-state dependent ArnAB complex formation. The phosphorylation state of ArnB at each time point of these pull downs was confirmed by LC–MS/MS after tryptic digestion. No ArnC was observed in pull-downs.

Phosphorylated ArnB was prepared by using the plasmid coding for ArnB alone (Supplementary Figure S15). For HDX-MS samples, phosphorylated ArnB either alone or associated with ArnA was isolated by Ni–NTA affinity chromatography via the His₆-tag of ArnB, followed by size-exclusion chromatography to remove unbound proteins.

### 4.5 Crystallization and structure determination of the ArnAB complex

Freshly purified, equimolar protein of ArnA and ArnB (4.85 mg/mL) was filtered with a centrifuge reaction tube micro filter (Merck) prior to crystallization experiments. Initial crystal hits of the ArnAB complex grew in well F11 of the JCSG Core III Suite (NeXtal) but were unsuitable for structure determination. Accordingly, a fine screen hanging drop vapor diffusion experiment on 24-well plates by mixing 1 μL of crystallization condition with 1 μL protein solution was conducted. The final monoclinic crystals grew in the presence of 0.17 M ammonium acetate, 0.085 M sodium citrate pH 6.0, 25.5% (w/v) PEG 4000, 16.5% (v/v) glycerin after 5 months.

ArnAB data set was collected at the Swiss Light Source at Paul Scherrer Institute (*Switzerland*). Due to anisotropic diffraction (highest resolution 2.11 Å, spherical completeness 2.54 Å), X-ray data were integrated by XDS (Kabsch, 2010) and scaled with the STARANISO server (Supplementary Table S3, Supplementary Dataset), Structure determination of the ArnAB complex was done by molecular replacement with the ArnE (vWA2) structure (PDB: 5A8J) from *Sulfolobus acidocaldarius* as search model using Phaser (McCoy et al., 2007). Afterwards, the structure was refined by multiple rounds of manual structure building with Coot (Emsley et al., 2010) followed by phenix.refine (Afonine et al., 2012), which also led to electron density defining the ArnA ZnF-domain. Data collection and refinement statistics can be found in Supplementary Table S2. Protein structure was deposited to the Protein Data Base and can be accessed via the code: 8S05.

### 4.6 Small angle X-ray scattering (SAXS) of ArnAB complex

SAXS datasets were collected at the synchrotron Bio-SAXS beamline BM29 (ESRF) Grenoble, France (Pernot et al., 2013). The wavelength *λ* = 1.0 Å and the sample-to-detector distance of 2.43 m resulted in scattering vectors, *q*, ranging from 0.0025 Å^−1^ to 0.50 Å^−1^. The scattering vector is defined as *q* = 4π sin*θ*/*λ*, where 2*θ* is the scattering angle. All experiments were performed at 20 °C and the data were initially processed by the ATSAS software package (Petoukhov et al., 2012). SAXS data collection was performed in 150 mM NaCl, 50 mM Tris (pH 8) buffer. One-dimensional datasets were subtracted from the buffer-only spectrum then merged and analyzed in Primus (Konarev et al., 2003). 1D-scattering of different concentrations were merged, where appropriate, given that the lower concentrations better represent low *q* data points. The radius of gyration was calculated by ScÅtter (Tully et al., 2021). The *I*_0_-values were calculated considering bovine serum albumin (BSA) as standard, where the *R*_g_ was 33.6 ± 0.4 Å and I_0_ 93.13 ± 0.1. *P*(*r*) distance distribution functions were calculated and refined by the program ScÅtter. The distance *r*, where the *P*(*r*) functions approach zero probability, identifies the maximal dimension (*D*_max_) of the macromolecule. Using the refined data from ScÅtter in GNOM-format *ab initio* models for the corresponding SAXS-derived electron densities were calculated by averaging 20 rounds of DENSS (Grant, 2018). These electron densities were fitted with structures and finally visualized by ChimeraX. The SAXS data sets are deposited in SASBDB (Valentini et al., 2015) with accession code SASDUW2.

### 4.7 Hydrogen/deuterium exchange mass spectrometry (HDX-MS) of ArnAB complex

Investigated proteins (ArnA, ArnB, ArnB~P, ArnA/ArnB~P) were employed as 50 μM concentrated stocks solutions in 20 mM Tris-Cl pH 8.0, 150 mM NaCl. From these, HDX reactions were prepared by an autosampler (LEAP Technologies) as follows: 6.5 μL of protein stock solution were pre-dispensed in a 96-well plate, 58.5 μL of a buffer (20 mM Tris-Cl pH 8.0, 150 mM NaCl) prepared with 99.9% D_2_O added and incubated at 25 °C for 10/30/100/1,000/10,000 s. The HDX reaction was stopped by transferring 55 μL of the reaction to 55 μL of quench solution [400 mM KH_2_PO_4_/H_3_PO_4_, 2 M guanidine-HCl (pH 2.2) pre-dispensed in another 96-well plate cooled down to 1 °C]. 95 μL of the quenched reaction were injected into an ACQUITY UPLC M-Class system with HDX Technology (Waters) operating at 0.5 °C through a 50-μL injection loop. Non-deuterated samples were generated analogously with an H_2_O-based buffer.

Samples were washed out of the loop with water + 0.1% (v/v) formic acid (100 μL/min) and digested at 12 °C in a cartridge (2 cm × 2 mm) filled with either bead-immobilized porcine pepsin or a 1:1 mixture of bead-immobilized protease type XVIII from *Rhizopus* sp. and protease type XIII from *Aspergillus saitoi*. The resulting peptides were collected on a trap column (2 mm × 2 cm; 0.5 °C) filled with POROS 20 R2 reversed phase resin (ThermoFisher Scientific). After 3 min of digestion and trapping, the trap was placed in line with an ACQUITY UPLC BEH C18 1.7 μm 1.0 × 100 mm column (Waters) operating at 0.5 °C and the peptides eluted with a gradient of water + 0.1% (v/v) formic acid (eluent A) and acetonitrile + 0.1% (v/v) formic acid (eluent B) at 30 μL/min, as follows: 0–7 min/95–65% A, 7–8 min/65–15% A, 8–10 min/15% A. The peptides were guided to a Synapt G2-Si mass spectrometer (Waters) and ionized by electrospray ionization (capillary temperature: 250 °C; spray voltage: 3.0 kV). Mass spectra were acquired with MassLynX MS 4.1 (Waters) over 50–2,000 *m/z* in enhanced high-definition MS (HDMS^E^) (Geromanos et al., 2009; Li et al., 2009) or high-definition MS (HDMS) mode for non-deuterated and deuterated samples, respectively. A short spray of [Glu1]-fibrinopeptide B standard (Waters) every 45 s was employed for lock-mass correction. During peptide separation, the protease column was washed three times with 80 μL of 0.5 M guanidine-HCl in 4% (v/v) acetonitrile, and blank runs (double-distilled H_2_O) were performed between each sample. All measurements were performed in triplicates for each protease column type (separate HDX reactions).

Peptides were identified ProteinLynx Global SERVER 3.0.1 (PLGS, Waters) and DynamX 3.0 (both Waters) from the non-deuterated samples acquired with HDMS^E^ as described previously (Joiner et al., 2023) employing low-energy, elevated-energy and intensity thresholds of 300, 100 and 1,000 counts, respectively and matched using a database containing the amino acid sequences of ArnA, ArnB, porcine pepsin, and their reversed sequences (peptide tolerance = automatic; fragment tolerance = automatic; min fragment ion matches per peptide = 1; min fragment ion matches per protein = 7; min peptide matches per protein = 3; maximum hits to return = 20; maximum protein mass = 250,000; primary digest reagent = non-specific; missed cleavages = 0; false discovery rate = 100). For quantification of deuterium incorporation with DynamX, peptides had to fulfil the following criteria: identification in two-thirds of the non-deuterated samples; minimum intensity of 30,000 counts; maximum length of 30 residues; minimum number of products of three and 0.1 products per residue; maximum mass error of 25 ppm; retention time tolerance of 0.5 min. Hereby, the datasets generated with porcine pepsin or after digestion with proteases type XIII and XVIII were pooled, and all spectra manually inspected. Residue-specific deuterium uptake of peptides was calculated with DynamX. If a residue was covered by a single peptide, the residue-specific deuterium uptake was equal to that of the whole peptide. In the case of overlapping peptides for a given residue, the residue-specific deuterium uptake was determined by the shortest peptide covering that residue. When multiple peptides were of the shortest length, the peptide with the residue closest to the C-terminus was utilized.

### 4.8 Growth and starvation assays of *Sulfolobus acidocaldarius* arnA and arnB mutants

*S. acidocaldarius* background strain MW001 (Wagner et al., 2012) and deletion strains MW351 (Δ*arnA*) and MW353 (Δ*arnB*) were grown in Brock Basic (Brock et al., 1972) media with 0.1% NZ-amine, 0.2% dextrin and 10 μg/mL uracil (rich medium), (pH 3.5), at 75 °C, 120 rpm to an OD_600_ of 0.5. Cells were starved as previously described (Bischof et al., 2019) by spinning down 200 mL culture (4,863*g). The cell pellet was resuspended in 200 mL preheated rich or starved medium (Brock Basic with uracil). The resuspended cultures were grown at 75 °C for 4 h. The cultures were centrifuged at 4863*g, 4 °C for 20 min. The cell pellet was frozen in liquid nitrogen and the dry cell mass was determined to be 300(±50) mg.

### 4.9 Proteomics of arnA and arnB deletion mutants

Sample preparation and measuring of the *S. acidocaldarius* proteomes was performed as previously described (Korf et al., 2023) in collaboration with the bioanalytic MarMass facility of the Philipps University of Marburg.

The timsTOF data was analyzed utilizing MaxQuant 2.1.3. Sequencing information for *S. acidocaldarius* DSM 639, comprising 2,222 entries, was retrieved from the UniProt database. For label-free quantification (LFQ) analysis, MaxQuant parameters were configured as follows: maximum peptide mass of 4,000 Da, allowance of up to three modifications per peptide, up to three missed cleavages, and the necessity of MS/MS for LFQ comparisons; all other parameters remained at default settings. Protein abundances from biological triplicates of the Δ*arnA*, Δ*arnB* and the wildtype strain were filtered using a two-tailed student’s *t*-test (*p* < 0.05). The remaining proteins were visualized in protein heat maps, with further filtering based on a mean abundance difference of >0.5 log_2_ fold-change. Visualization was done using the pheatmap R package (Kolde, 2025) in addition to the gplots package (Warnes et al., 2024). The volcano plots were constructed by plotting the log_2_ fold-change in protein abundance on the *x*-axis against the negative logarithm (base 10) of the *p*-values (calculated from the student’s *t*-test) on the ordinate. Plots were created using the ggplot2 (Wickham, 2016) and ggrepel (Slowikowski et al., 2024) packages in R, providing a comprehensive visualization of the proteomic changes in the context of significance levels. Gene Ontology (GO) enrichment analysis was performed on the sets of significant proteins (*p* < 0.05) from each comparison using GOATOOLS library version 1.2.3 (Klopfenstein et al., 2018). The significance of the GO enrichment analysis was determined by applying the Benjamini-Hochberg procedure with a false discovery rate (FDR) correction at a significance threshold of *p* = 0.05. The analysis was carried out with gene ontology terms specific to *S. acidocaldarius* DSM639, obtained via the QuickGO (Binns et al., 2009) annotation tool.

### 4.10 Mutagenesis of ArnB threonines

For point mutation of the different ArnB mutants 10–60 ng of starting vector was mixed with 150 ng of corresponding primers (Supplementary Table S1), 1.5 μL DMSO, 1 μL dNTP mix (NEB), 0.5 μL of Phusion DNA-polymerase stock (~6 mg/mL) and adjusted to 50 μL with H_2_O. The PCR was performed for 18 cycles, primer suitable annealing temperatures (50–56 °C) and an elongation temperature of 72 °C. PCR was followed by a *Dpn*I (2 μL, NEB) digest (37 °C, 2 h) and transformation into *E. coli* DH5α cells. Afterwards plasmids were extracted from overnight cultures using the QIAprep Spin Miniprep Kit (Qiagen) and checked by Sanger sequencing.

## Data Availability

The datasets presented in this study can be found in online repositories. The names of the repository/repositories and accession number(s) can be found in the article/Supplementary material. RCSB PDB entry 8S05 is available in database (https://www.rcsb.org/structure/8S05).
