# Supplementary material for: Phosphorylation-driven conformational switching of the ArnA–ArnB complex involved in archaeal motility regulation
Source: Front Microbiol. 2026 Jan 15;16:1717585. doi: 10.3389/fmicb.2025.1717585 (PMC12852372; doi:10.3389/fmicb.2025.1717585)
Supplement: Supplementary file 1 [file Data_Sheet_1.PDF]

Supplementary Material

Phosphorylation-Driven Conformational Switching of the ArnA-ArnB Complex Involved in Archaeal Motility Regulation

Mohamed Watad[1‡], Lukas Korf[1‡], Wieland Steinchen[1,2], Filipp Bezold1, Marian S. Vogt[1], Po Hsun Wang[1], Leon Selbach[1], Sebastian Hepp[1], Luis Gayermann[3], Marleen van Wolferen[3], Xing Ye[3], Sonja-Verena Albers[3,4*], Lars-Oliver Essen[1*]

[1]Department of Chemistry, Philipps University, Hans Meerwein-Str. 4, 35043 Marburg, Germany

[2]Center for Synthetic Microbiology, Philipps University Marburg, Karl-von-Frisch-Str. 14, 35043 Marburg, Germany

[3]University of Freiburg, Institute for Biology, Molecular Biology of Archaea, Schaenzlestrasse 1, 79104 Freiburg, Germany

[4]Signalling Research Centres BIOSS and CIBSS, University of Freiburg, 79104 Freiburg, Germany

[‡]Co-first authors Corresponding author

[*]Corresponding author

Email: essen@chemie.uni-marburg.de
sonja.albers@biologie.uni-freiburg.de

This PDF file includes:

Supplementary text
Figures S1 to S16
Table S1 to S3

Other supplementary materials for this manuscript include the following:

Supplementary Dataset 1 (HDX data)
Supplementary Dataset 2 (proteomics data Δ*arnA* und Δ*arnB*)
Supplementary Dataset 3 (proteomics data Δ*arnE*)
Supplementary Dataset 4 (Foldseek analysis)
Supplementary Dataset 5 (Phosphorylation MS)

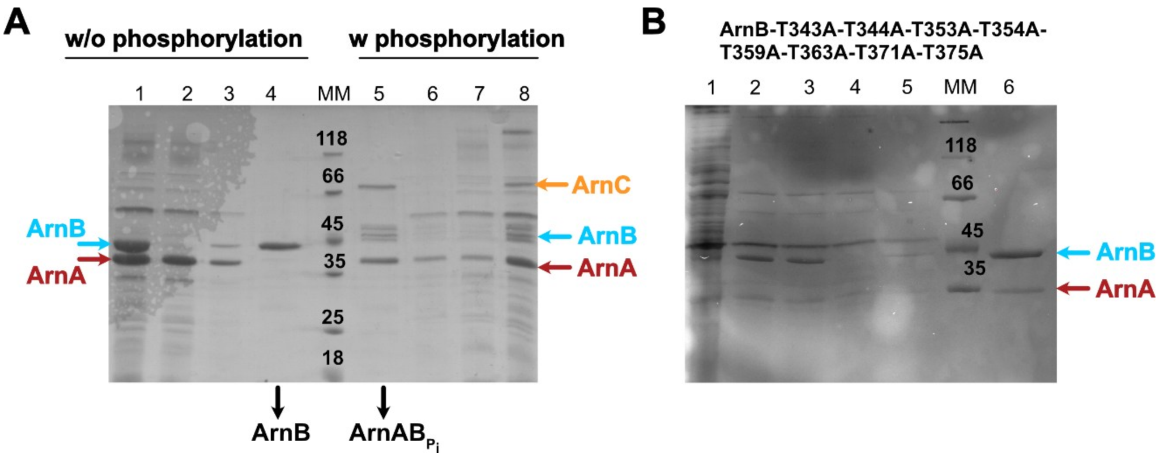

Figure S1: ArnA/ArnB pull-down assays. A: SDS-PAGE with a comparison of Ni-NTA column purifications of ArnA and ArnB without phosphorylation (left part, lanes 1-4) and with phosphorylation by ArnC (right side, lanes 5-8). ArnB is only able to pull-down ArnA when they were subjected to prior phosphorylation. 1: heat step, 2: flow through, 3: wash, 4: elution, MM: molecular marker, 5: elution, 6: wash, 7: flow through, 8: heat step/ P_i assay. **B:** Ni-NTA purification of the multiple Thr→Ala mutant of ArnB after 60 min phosphorylation by ArnC. Under these prolonged phosphorylation times this mutant of ArnB depleted of threonines in the α8/α9 helices of the RHB is still able to pull-down ArnA. 1: lysate, 2: heat step, 3: P_i assay, 4: flow through, 5: wash, 6: elution.

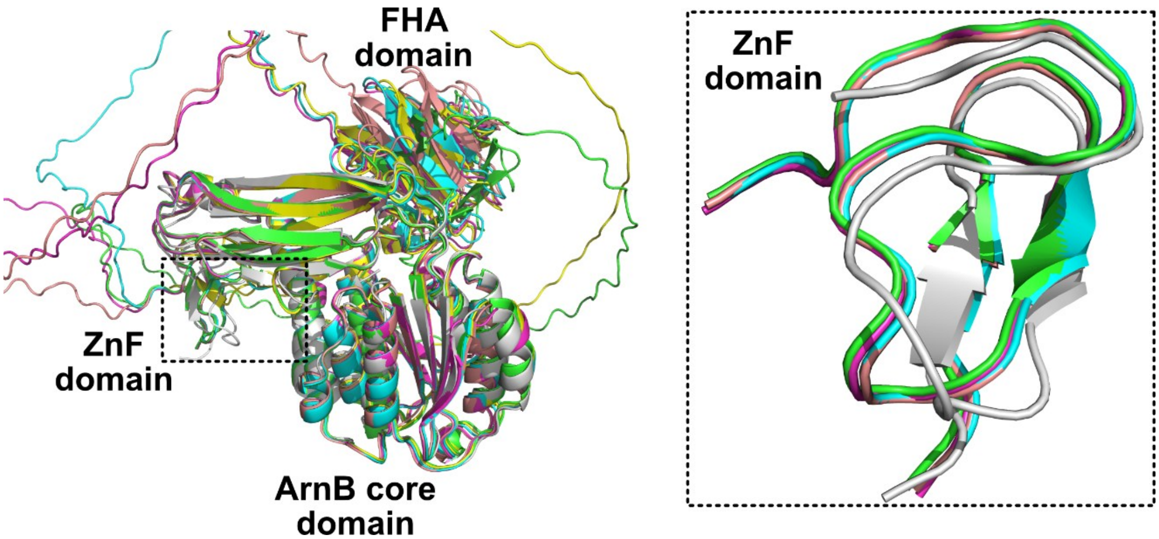

Figure S2: Superposition of experimental and predicted unphosphorylated ArnAB complex. Complexes for ArnAB were predicted by the AlphaFold2-Multimer pipeline and superimposed to the ArnAB crystal structure (8S05). *Left*, Structural alignment showing the five models generated by AF2. Disordered regions of the AF2 models correspond to the linker region between the ZnF and FHA domains of ArnA. *Right*, detail view on the ZnF domains in the ArnAB complex showing only minor deviations from the ArnAB cocrystal structure (gray). The five AF2 models of the ArnAB complex are colored according to their rank in green, cyan, pink, yellow and salmon.

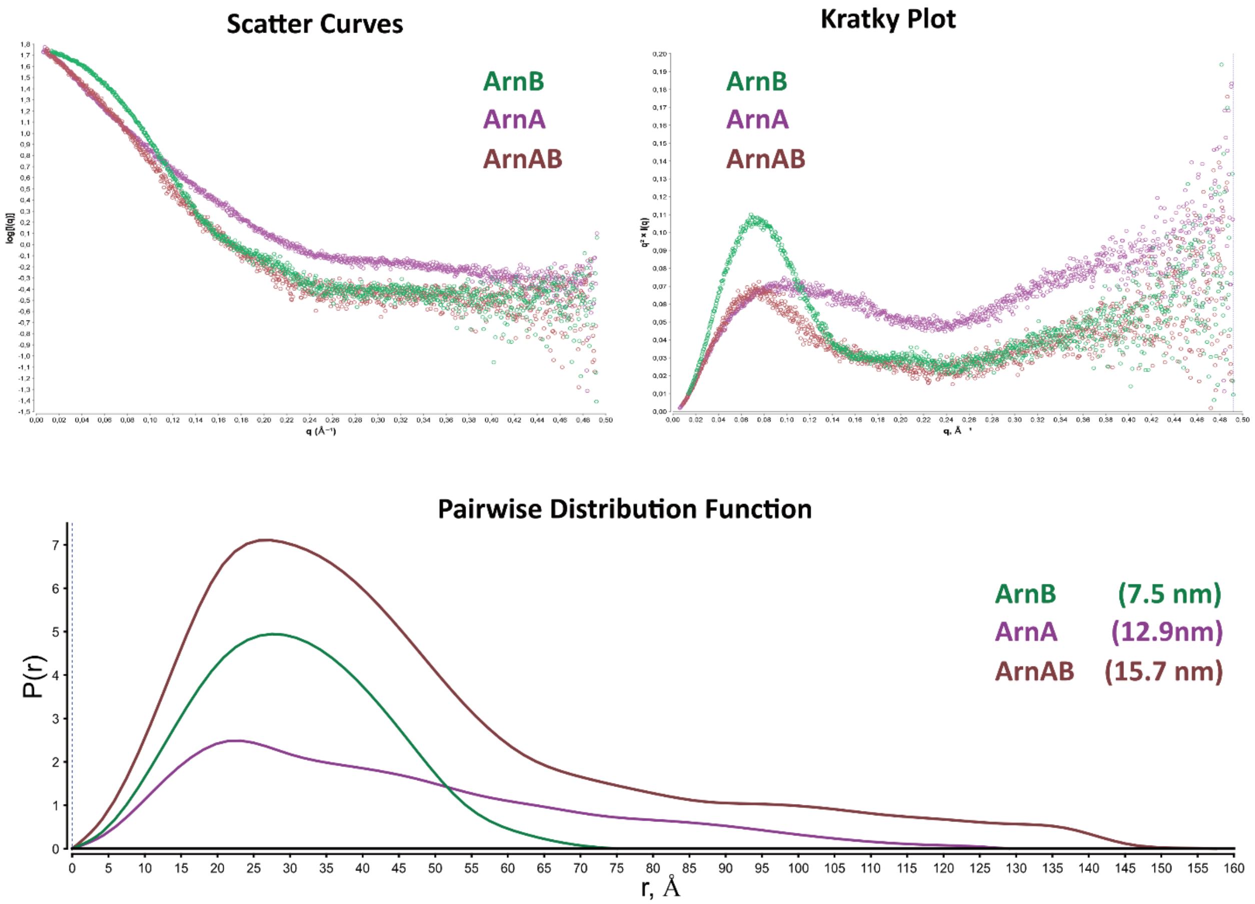

Figure S3: SAXS data analysis of ArnA, ArnB and the ArnAB complex. with. The representations for the scatter curves, Kratky plot and pairwise distribution function show the average of technical triplicates for each of the three SAXS samples: unphosphorylated ArnAB (brown), ArnA (purple) and ArnB (green) with D_{max} shown in parentheses.

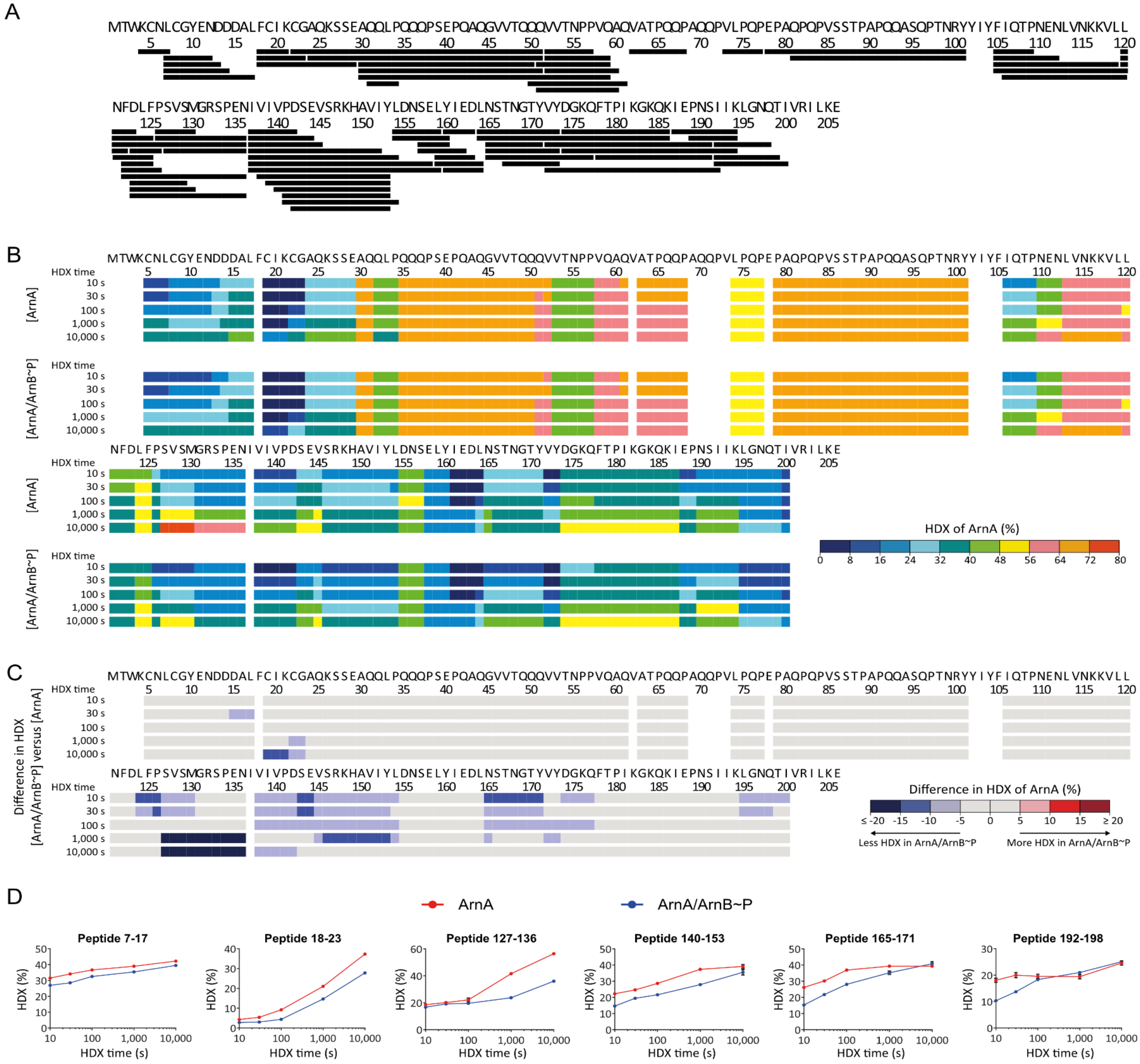

Figure S4: HDX data evaluation of ArnA. A: Each black bar represents a peptide of ArnA identified in HDX-MS. **B:** The residue-specific HDX of individual ArnA and that of ArnA in complex with ArnB~P (ArnA/ArnB~P) is projected onto the ArnA amino acid sequence. **C**: The difference in residue-specific HDX between ArnA in complex with ArnB~P and individual ArnA is projected onto the ArnA amino acid sequence. Blue color denotes reduced HDX of ArnA when in complex with ArnB~P. **D**: Progression of HDX over time for selected ArnA peptides from either ArnA (red) or ArnA/ArnB~P (blue) samples. Data represent mean ± s.d. of n = 3 technical replicates (individual HDX reactions).

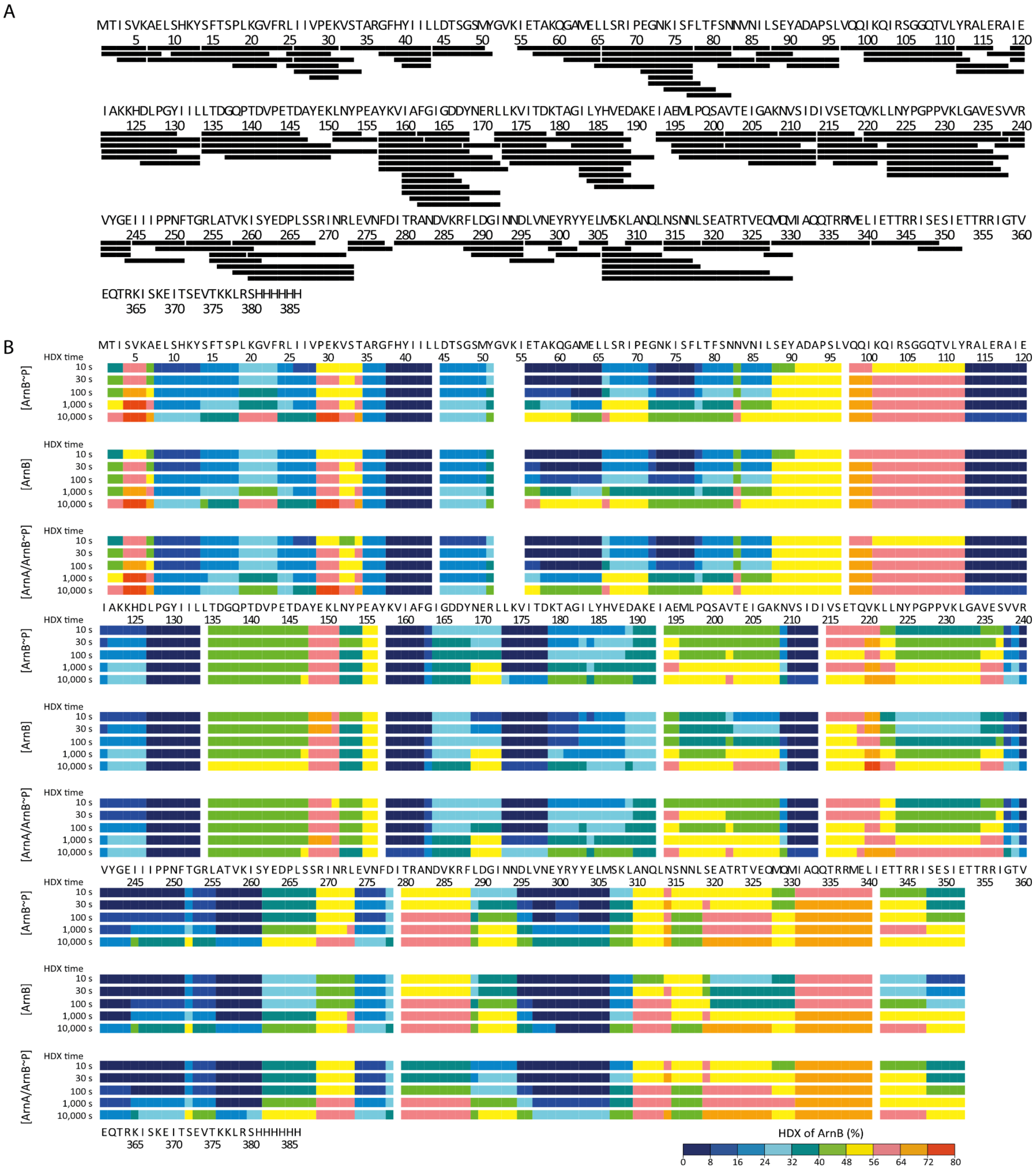

Figure S5: HDX data evaluation of phosphorylated ArnB and phosphorylated ArnAB complex. A: Each black bar represents a peptide of ArnB identified in HDX-MS. **B:** The residue-specific HDX of ArnB, ArnB~P and that of ArnB~P in complex with ArnA is projected onto the ArnB amino acid sequence.

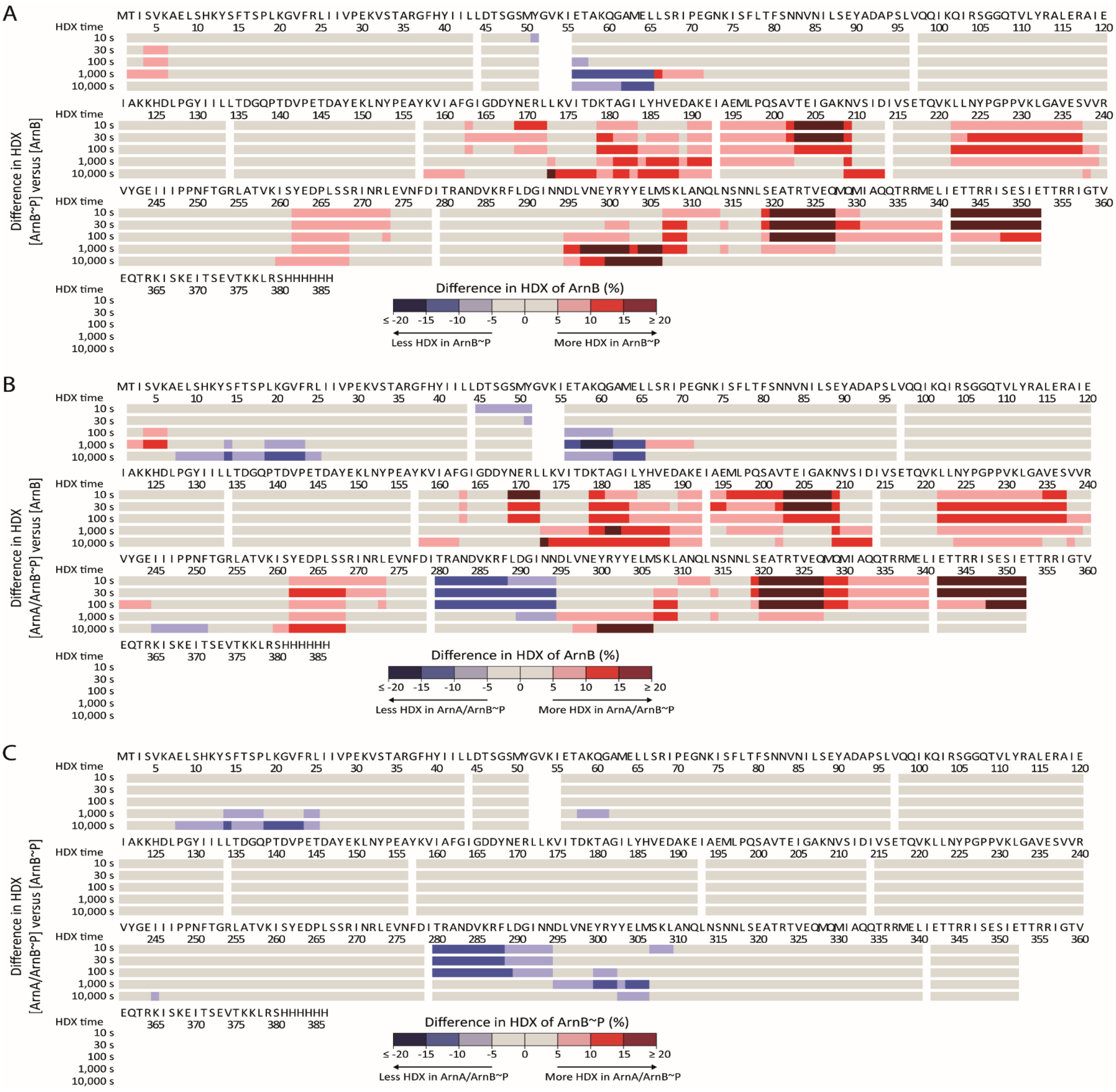

Figure S6: HDX data evaluation of the impact of phosphorylation on the interaction of ArnA and ArnB. A-C: The difference in residue-specific HDX between ArnB~P and ArnB (**A**), ArnA/ArnB~P and ArnB (**B**), as well as ArnA/ArnB~P and ArnB~P (**C**) is projected onto the ArnB amino acid sequence. Red color reflects elevated HDX of ArnB upon phosphorylation (A and B), and blue color reflects reduced HDX upon phosphorylation (A and B) and ArnA binding (B and C).

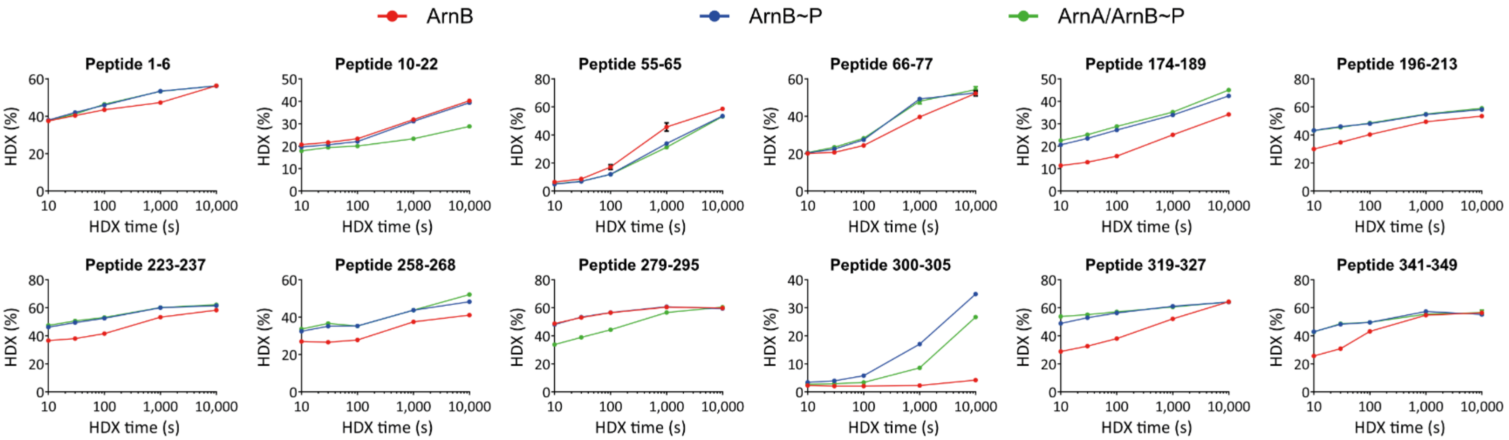

Figure S7: HDX MS of ArnA and ArnB peptides. Progression of HDX over time for selected representative ArnB peptides derived from ArnB (red), ArnB~P (blue) and ArnA/ArnB~P (green) samples. Data represent mean ± s.d. of n = 3 technical replicates (individual HDX reactions).

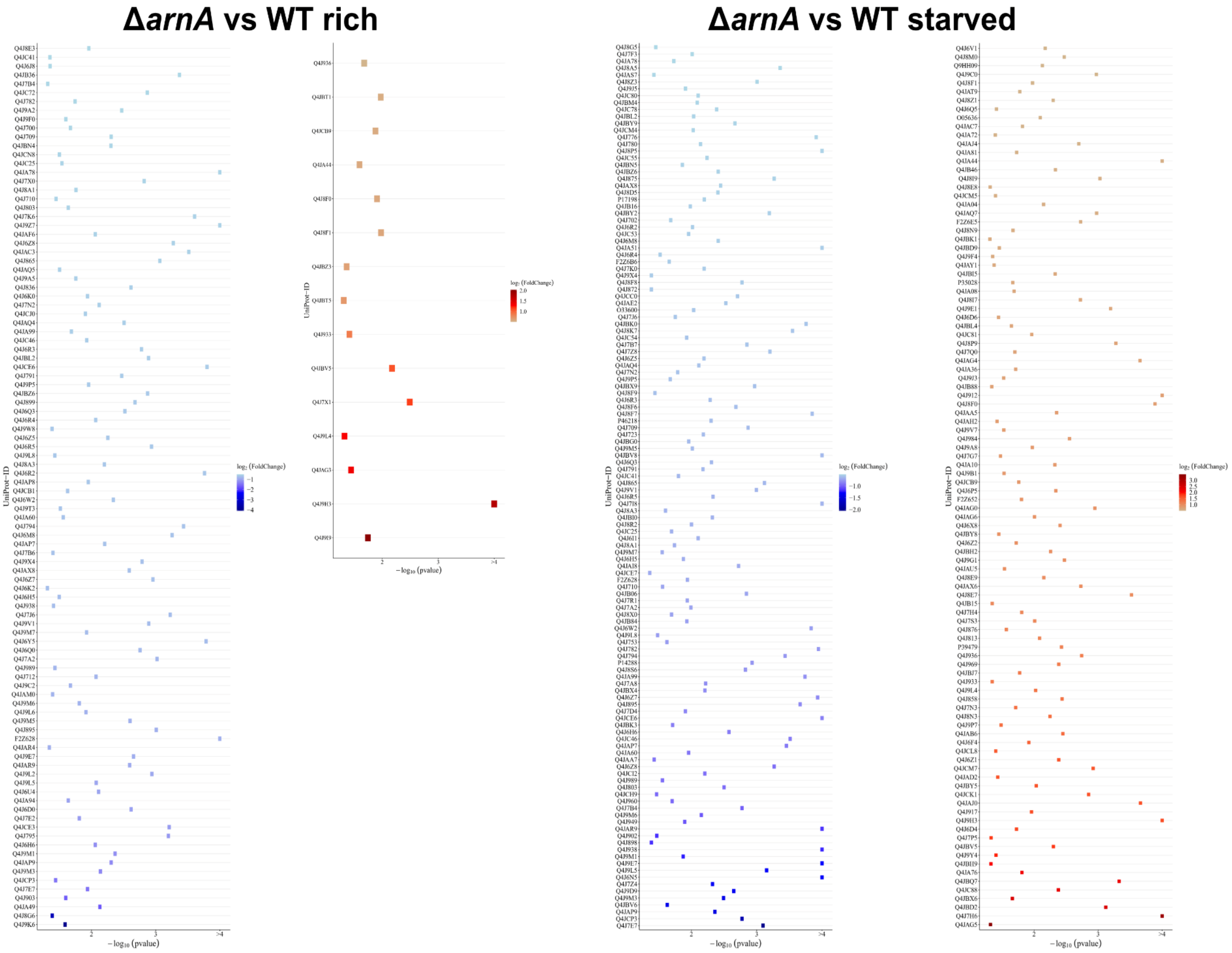

Figure S8: Volcano plot analysis of Δ*arnA* vs. WT showing Uniprot IDs of over- (red) or under-regulated (blue) proteins under the indicated conditions. Only proteins with a significance level of at least $p<0.05$ and a twofold change were considered. The analysis charts do refer to the indicated knockout strain, i.e. which proteins were up- or down-regulated in the knockout strain in comparison to WT. Higher significant levels are shown towards the right, whereas the degree of changes are ranked from the top to the bottom.

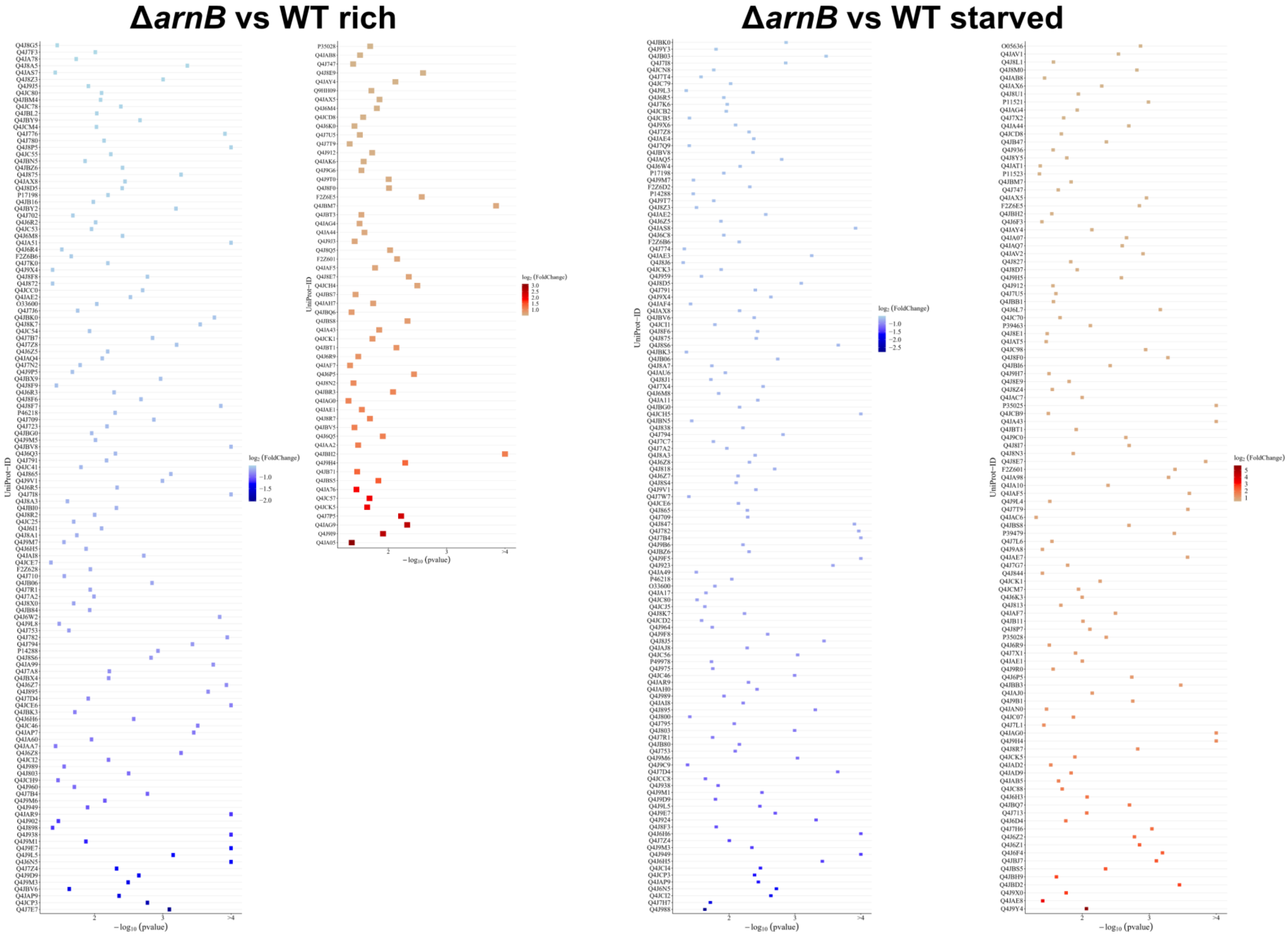

Figure S9: Volcano plot analysis of Δ*arnB* vs. WT showing Uniprot IDs of over- (red) or under-regulated (blue) proteins under the indicated conditions. Only proteins with a significance level of at least p<0.05 and a change of at least 50% were considered. The analysis charts do always refer to the knockout strain, i.e. which proteins were up- or down-regulated relative to the WT; significance levels increase from left to right, level changes from top to bottom. Some of the proteins with minor down-regulated changes under starved conditions were cut off for simplicity but are listed in the proteomics Table S3.

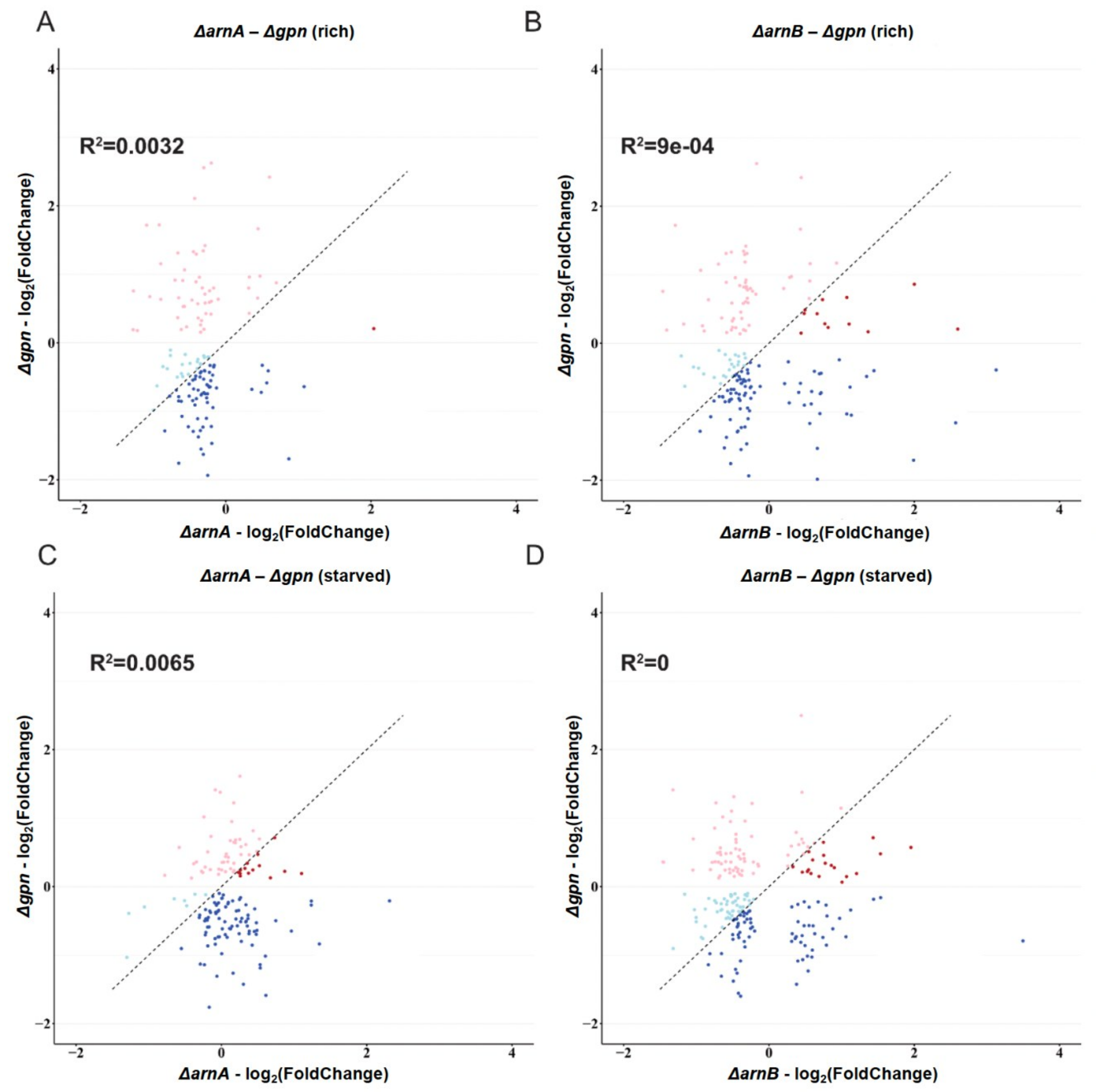

Figure S10: Proteomic changes are independent in *Sa*GPN and *arnA*/*arnB* knockout strains. A: Correlation plot of the *S. acidocaldarius* Δ*arnA* vs. Δ*gpn* deletion strains under nutrient-rich conditions, revealing very weak correlation. **B:** Correlation plot of the *S. acidocaldarius* Δ*arnB* vs. Δ*gpn* deletion strains under nutrient-rich conditions. **C:** *S. acidocaldarius* Δ*arnA* vs. Δ*gpn* deletion strains under nutrient starvation. **D:** *S. acidocaldarius* Δ*arnB* vs. Δ*gpn* deletion strains under nutrient starvation.

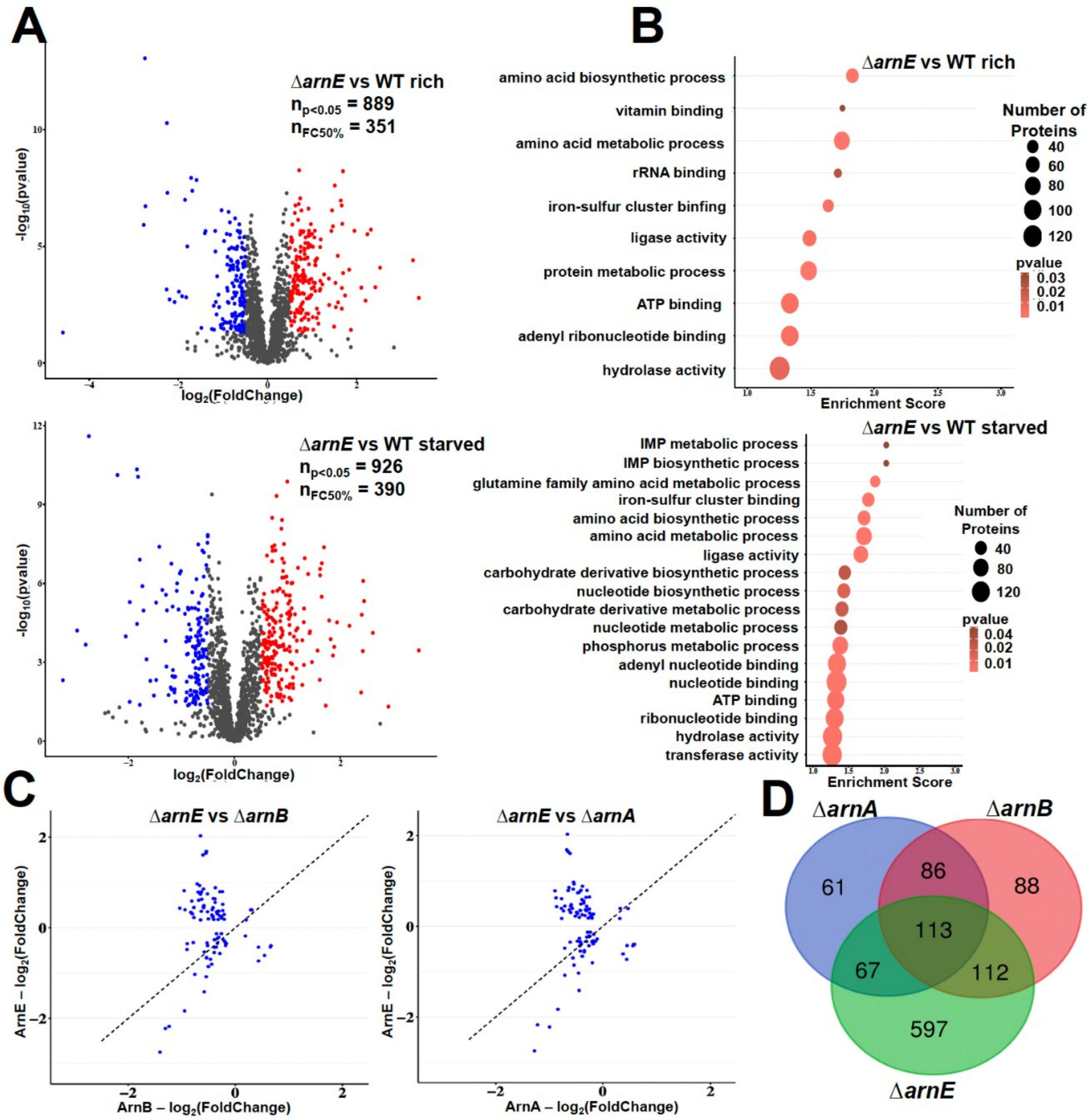

Figure S11: Proteomic analysis of *Sulfolobus acidocaldarius* Δ*arnE* strain. A: Proteomic analyses of Δ*arnE* strain vs. wild type strain visualized as volcano plots in respect to the corresponding nutritional state. The top plot shows nutrient-rich conditions, the bottom plot nutrient-starved conditions. Sample size consists of three biological replicates per condition, as well as two technical duplicates per measurement. Identified gene product levels with p-values < 0.05 and log_2 fold change >0.5 are colored, indicating downregulation (blue) in the Δ*arnE* strain or upregulation (red). **B**: Gene enrichment analysis of identified hits with clustering into GO terms reveals mostly metabolic processes like amino acid metabolism, nucleotide biosynthesis, carbohydrate metabolism, ribosomes, ATP and iron-sulfur cluster-dependent activities. **C**: Scatter plots of gene products commonly found in Δ*arnE* vs. Δ*arnA* or Δ*arnB* strains highlight a mix of shared and non-shared correlations, suggesting both cooperative and distinct functions of ArnE compared to ArnA and ArnB. **D**: Venn diagrams for the overlap between identified gene products (p

< 0.05) in Δ*arnE*, Δ*arnA* and Δ*arnB S. acidocaldarius* strains, showing that a core set of proteins is commonly regulated by the ArnAB complex (arnA/arnB: 61%/50%), while ArnE affects significantly less common changes of 20%/25% with arnA/arnB.

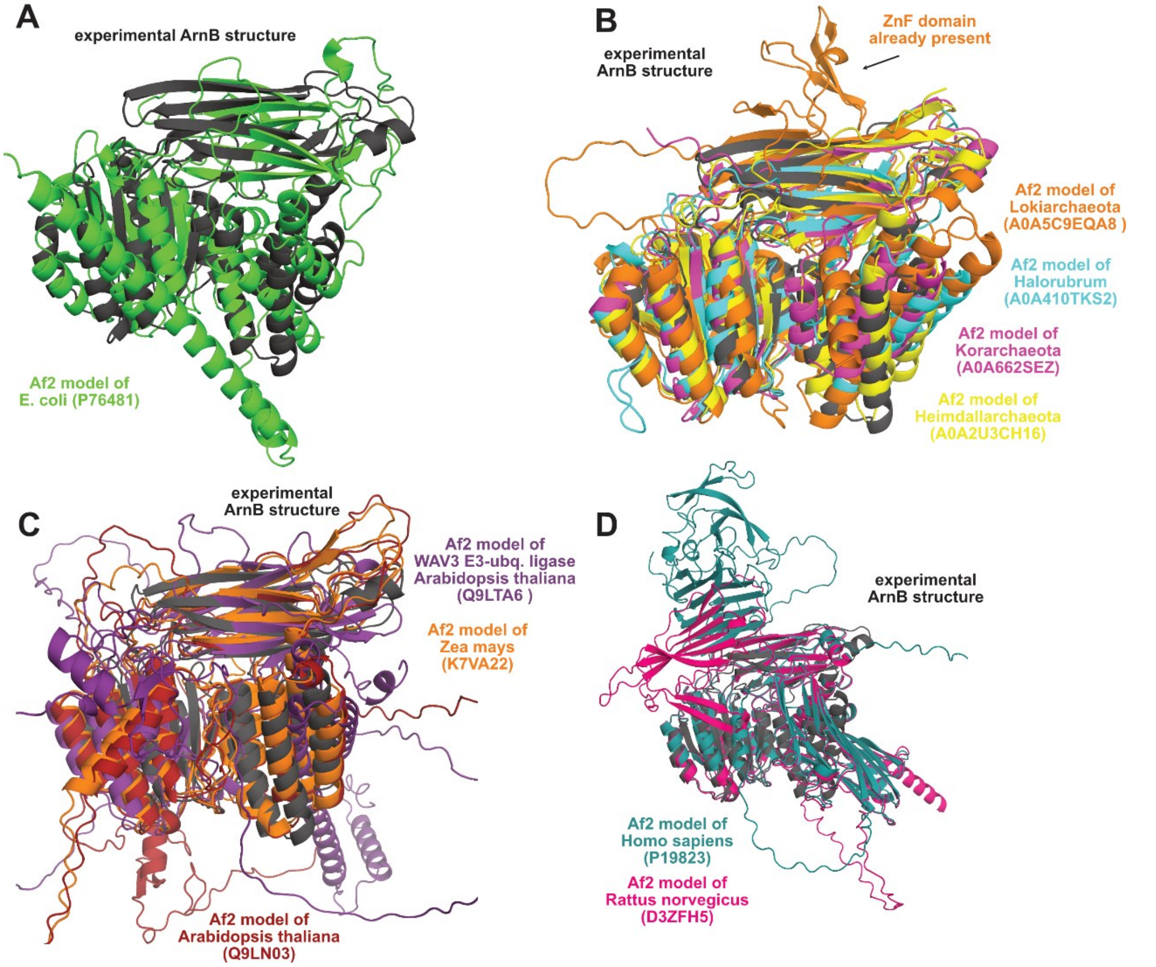

Figure S12: Structural conservation of the Sec23/Sec24 core motif and the ArnB structure. The ArnB crystal structure was subjected to foldseek search; selected hits as predicted by AlphaFold2 were superimposed to the ArnB structure that is representative of the Sec23/24 core motif. **A:** For the bacterial kingdom, the superposition of YfbK (Uniprot entry P76481) is shown. **B:** Archaeal orthologs from *Lokiarchaeota*, *Haloarchaea*, *Korarchaeota* and *Asgard* archaea. Interestingly, the member of the *Lokiarchaeota* is predicted to include a Sec23/24-like ZnF domain at an analogous position. **C:** Members of the plant family not belonging to the Sec23/Sec24-family show a high degree of structural conservation of the Sec23/Sec24 core motif. In contrast, other animal hits by foldseek (**D**) are predicted to be unrelated in terms of their domain packing. Uniprot identifiers are given in parentheses.

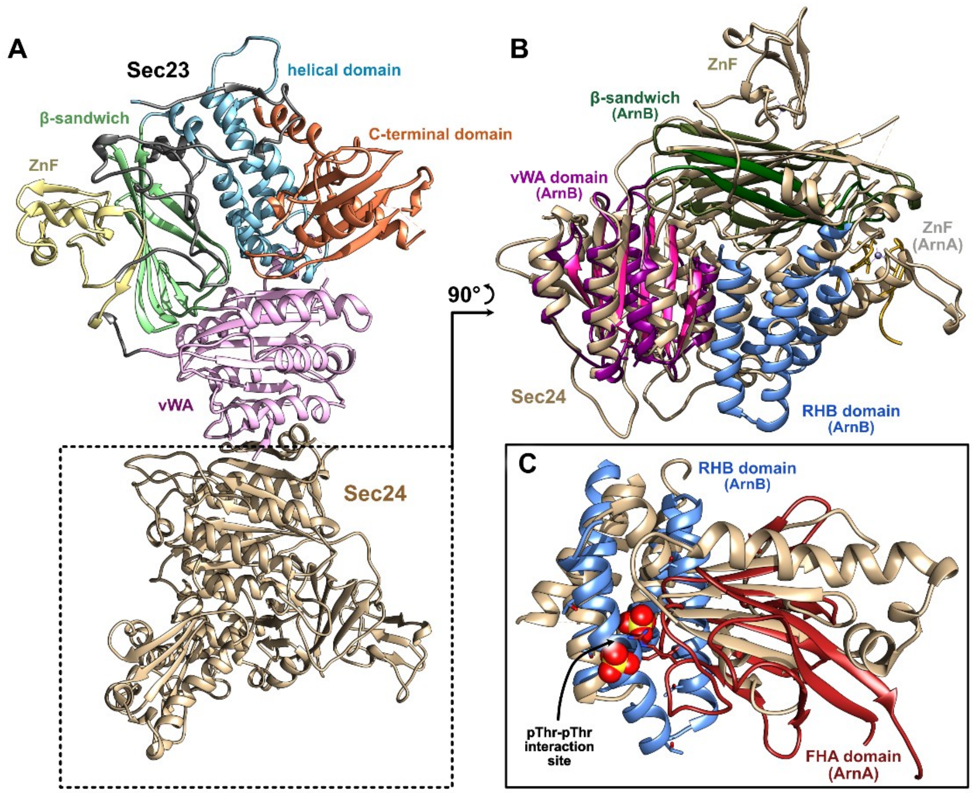

Figure S13: Structural homology between ArnB and the Sec23/Sec24 components of the COPII main membrane curvature factor. **A**: Domain arrangement of the Sec23/24 complex (PDB code: 1M2V). **B**: Superposition of Sec23/24 with ArnB without the C-terminal domain of Sec23/24, showing a high degree of alignment of the topological features. Structural similarity between ArnB and the Sec24 core motif (P301-I749 of chain B), 5.7 Å for 302 Cα atoms, is limited due to different helix lengths of the RHB's α8/α9 pair and the relative orientation of the β-sandwich. **C**: Superposition of ArnA FHA domain onto the C-terminal domain of Sec23/24 reveals structural similarities and aligns with a potential pThr-dependent interacting in a respective ArnAB complex.

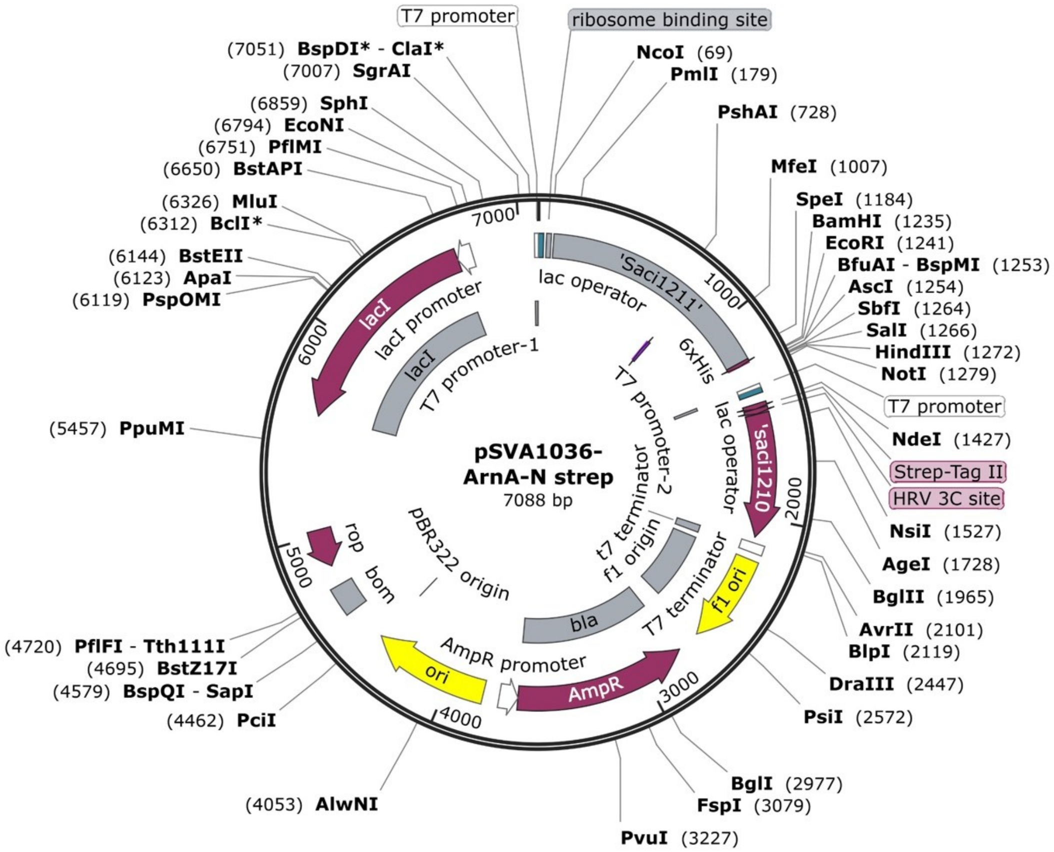

Figure S14: Plasmid map of pSVA1036-ArnA-N strep (7088 bp). The plasmid encodes the *Sulfolobus acidocaldarius* proteins ArnB (Saci_1211) and ArnA (Saci_1210) under control of two T7 promoter and lac operator system. ArnB carries a C-terminal 6×His tag, enabling purification via Ni-NTA affinity chromatography. ArnA is expressed with an N-terminal Strep-Tag II and HRV 3C protease cleavage site. The construct was used for co-expression and purification of the phosphorylated ArnAB complex employed in pull-down assays. The plasmid backbone carries an ampicillin resistance gene cassette (AmpR) for selection in *E. coli*. A multiple cloning site with numerous restriction sites (e.g., *Nco*I, *Bam*HI, *Eco*RI, *Hind*III, *Not*I, *Bgl*II) flanks the expression cassette.

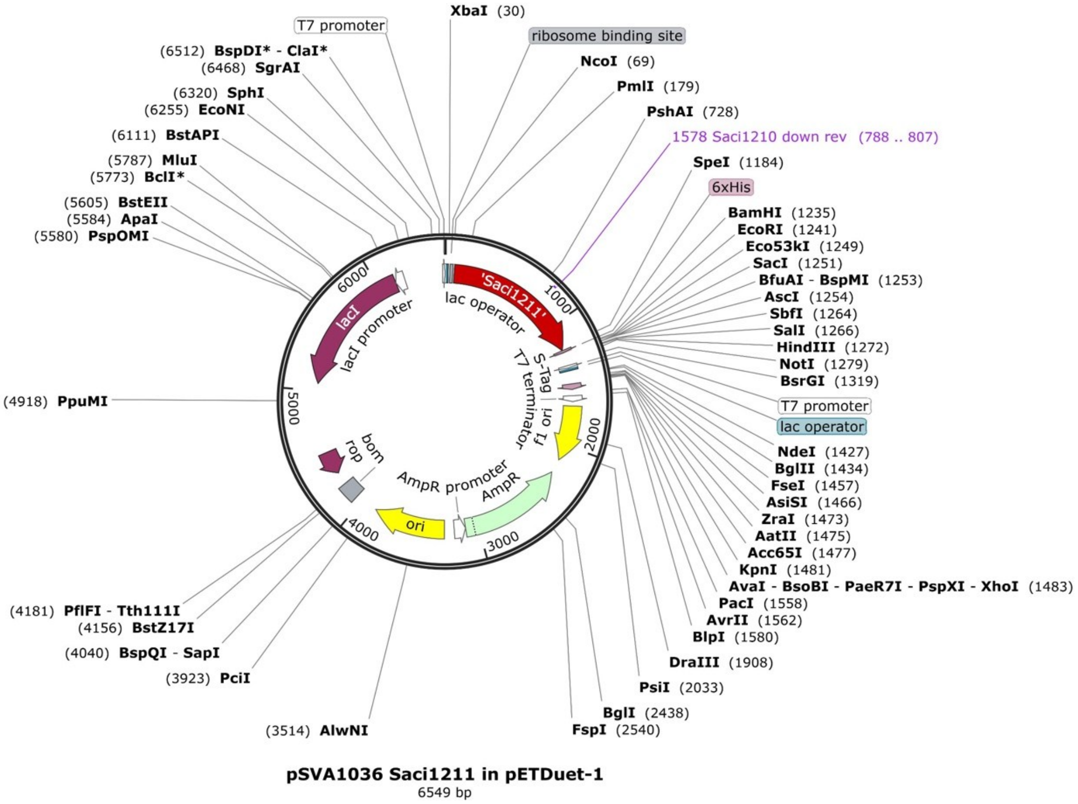

Figure S15: Plasmid map of pSVA1036 with ArnB alone in pETDuet-1 (6549 bp). This plasmid encodes ArnB (Saci_1211) from *Sulfolobus acidocaldarius* under control of a T7 promoter and lac operator system. ArnB carries a C-terminal 6×His tag for Ni-NTA affinity purification. The plasmid contains an ampicillin resistance gene cassette (AmpR) for selection in *E. coli*. The construct was used for recombinant overproduction of ArnB in *E. coli* BL21(DE3) Rosetta cells and subsequent purification by heat treatment, Ni-NTA, and SEC.

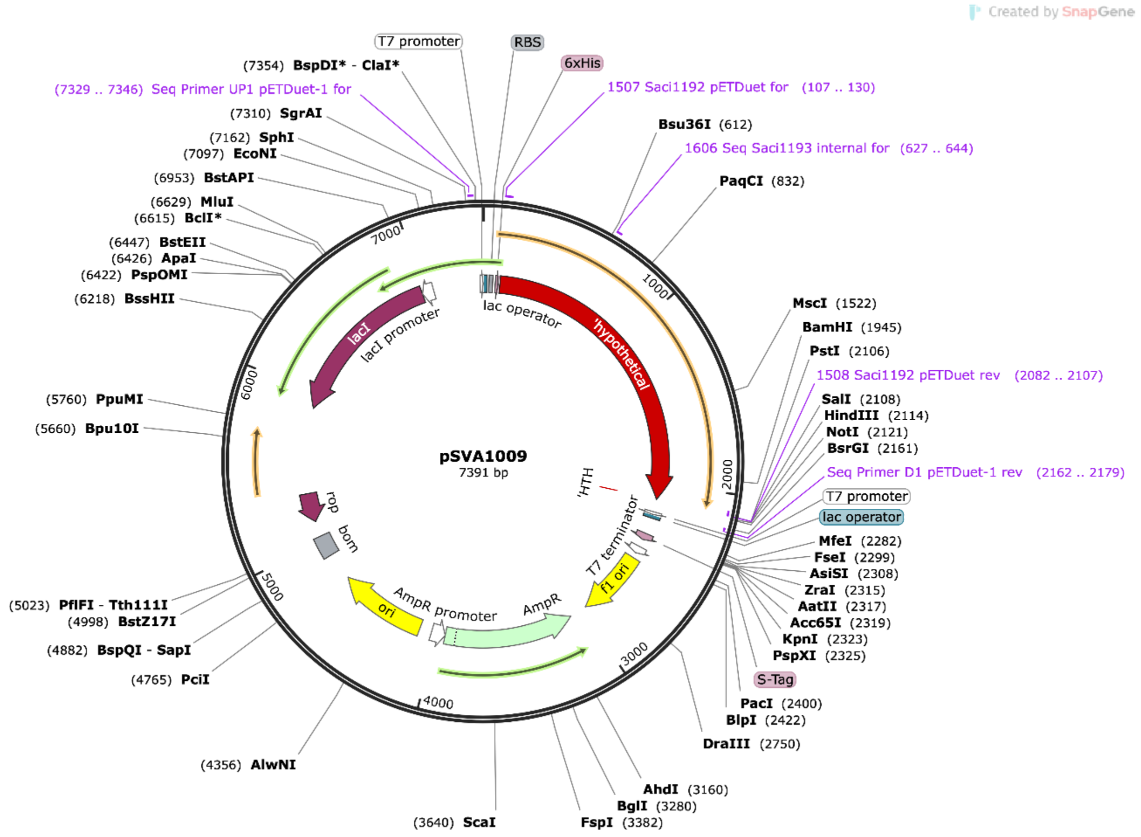

Figure S16: Plasmid map of pSVA1009 (7391 bp) for ArnC. This plasmid encodes ArnC (Saci_1193) from *Sulfolobus acidocaldarius* under control of a T7 promoter and lac operator system. The plasmid contains an ampicillin resistance gene cassette (AmpR) for selection in *E. coli*. A mutant without N-terminal His$_6$-tag was used for phosphorylation of ArnB and ArnAB after cell lysis, but prior to purification.

Table S1: Primers used for the side directed mutagenesis. Primers were used on the pETDuet^{TM}-1 coexpression vector encoding both *arnB-His$_6$* and *Strep-arnA*.

Primer Sequence	Introduced Mutation
CAGTAGAGCAAGCTAGAAAGATATCTAAGG	T363A, fw
CCTTAGATATCTTTCTAGCTTGCTCTACTG	T363A, rev
GAAGAATAGGTGCAGTAGAGCAAAC	T359A, fw
GTTTGCTCTACTGCACCTATTCTTC	T359A, rev
CGAAAGTATAGAGGCAGCTAGAAGAATAGG	T353A_T354A, fw
CCTATTCTTCTAGCTGCCTCTATACTTTCG	T353A_T354A, rev
GCTAATAGAGGCAGCTAGAAGAATAAGCG	T343A_T344A,fw
CGCTTATTCTTCTAGCTGCCTCTATTAGC	T343A_T344A, rev
CTAGTGAAGTTGCTAAGAAGTTGAGG	T375A, fw
CCTCAACTTCTTAGCAACTTCACTAG	T375A, rev
CTAAGGAAATAGCTAGTGAAGTTACTAAG	T371A, fw
CTTAGTAACTTCACTAGCTATTTCCTTAG	T371A, rev

Table S2: Crystallographic table for unphosphorylated ArnAB complex (8S05)

**Data collection and processing**	
X-ray source, beamline	SLS, Beamline X06SA
Detector	DECTRIS EIGER X 16M
Wavelength (Å)	1.00000
Space group	*I* 1 2 1
Cell dimensions (a, b, c,	50.1 Å, 147.4 Å, 62.6 Å
α, β, γ)	90.00°, 107.16°, 90.00°
Resolution (Å)	49.14 - 2.11 (2.36 - 2.11)
Total observations	92895 (3258)
Multiplicity	3.4 (2.4)
Unique reflections	27478 (1375)
Completeness, spherical (%)†	55.1 (9.7)
Completeness, ellipsoidal (%)†	89.9 (52.3)
R_{merge}	0.079 (0.505)
CC1/2	0.996 (0.638)
Mean I/σ(I)	9.2 (1.8)
Wilson *B*-factor (Å^2)	36.73
**Refinement**	
Resolution (Å)	49.14 - 2.11 (2.36 - 2.11)
R_{work}, R_{free}	0.2087 (0.3488), 0.2634 (0.2591)
Reflections (test set)	27435 (1363)
r.m.s.d. from ideal:	
Bond lengths (Å)	0.009
Bond angles (°)	1.10
Total number of atoms	6473
solvent	29
ligands	4
Average *B*-factor (Å^2)	48.29
Ligands	59.11
Ramachandran favored (%)	97.51
Ramachandran allowed (%)	2.11
Ramachandran outliers (%)	0.37

Statistics for the highest resolution shell are shown in parentheses.

† Dataset was corrected by STARANISO# for anisotropic diffraction. Cut-offs used direction -0.058 a* + 0.994 b* + 0.088 c* for best diffraction (2.11 Å), 0.588 a* + 0.809 c* for worst diffraction (3.54 Å). # Tickle, I.J., Flensburg, C., Keller, P., Paciorek, W., Sharff, A., Vonrhein, C., Bricogne, G. (2016). STARANISO, http://staraniso.globalphasing.org/cgi-bin/staraniso.cgi. Cambridge, United Kingdom: Global Phasing Ltd.

Table S3: STARANISO Data for X-ray crystallographic dataset of ArnAB complex.

									Compl. Spher.	*Compl. Ellip.*	*Multiplicity*	
Resolution		**#uniq**	**#obs**	**Rmerge**	**Rmeas**	**Rpim**	**#Isig**	**I/sigI**	**all**	**all**	**all**	**CC(1/2)**
(lower)	(upper)	--------	--------	---------	--------	-------	--------	--------	-------	-------	-----	--------
49.138	6.986	1373	4621	0.040	0.047	0.025	1373	24.182	0.974	0.974	3.39	0.997
6.986	5.538	1374	4840	0.050	0.059	0.031	1374	18.201	0.982	0.982	3.54	0.996
5.538	4.834	1373	5003	0.051	0.059	0.031	1373	19.308	0.989	0.989	3.65	0.996
4.834	4.389	1374	4393	0.053	0.064	0.035	1374	18.254	0.963	0.963	3.22	0.995
4.389	4.072	1375	4516	0.057	0.068	0.037	1375	16.543	0.976	0.976	3.32	0.995
4.072	3.830	1374	4704	0.070	0.083	0.044	1374	13.754	0.986	0.986	3.44	0.995
3.830	3.636	1372	4778	0.085	0.101	0.054	1372	11.389	0.984	0.984	3.49	0.994
3.636	3.479	1374	4828	0.102	0.121	0.064	1374	9.898	0.988	0.988	3.53	0.990
3.479	3.343	1375	4931	0.125	0.148	0.078	1375	8.594	0.979	0.979	3.60	0.989
3.343	3.224	1373	4936	0.153	0.181	0.095	1373	7.001	0.946	0.949	3.61	0.986
3.224	3.109	1374	4765	0.177	0.210	0.112	1374	6.129	0.859	0.922	3.49	0.978
3.109	3.002	1375	4329	0.182	0.221	0.122	1375	5.492	0.799	0.914	3.18	0.972
3.002	2.902	1374	4605	0.216	0.257	0.138	1374	4.958	0.739	0.902	3.37	0.965
2.902	2.807	1374	4719	0.258	0.306	0.162	1374	4.300	0.683	0.899	3.45	0.944
2.807	2.716	1374	4738	0.313	0.372	0.197	1374	3.532	0.628	0.908	3.46	0.927
2.716	2.627	1374	4811	0.394	0.468	0.248	1374	2.996	0.563	0.883	3.51	0.876
2.627	2.540	1374	4848	0.451	0.534	0.283	1374	2.687	0.500	0.879	3.54	0.840
2.540	2.453	1374	4848	0.510	0.603	0.318	1374	2.508	0.433	0.870	3.54	0.777
2.453	2.359	1373	4424	0.578	0.695	0.380	1373	2.032	0.349	0.820	3.24	0.690
2.359	2.109	1375	3258	0.505	0.659	0.416	1375	1.811	0.097	0.523	2.41	0.638
total	2.109	27478	92895	0.079	0.095	0.051	27478	9.178	0.551	0.899	3.40	0.996
